# Design, Synthesis, and Antitumor Evaluation of an Opioid Growth Factor Bioconjugate Targeting Pancreatic Ductal Adenocarcinoma

**DOI:** 10.3390/pharmaceutics16020283

**Published:** 2024-02-16

**Authors:** Justyna Budka, Dawid Debowski, Shaoshan Mai, Magdalena Narajczyk, Stanislaw Hac, Krzysztof Rolka, Eirinaios I. Vrettos, Andreas G. Tzakos, Iwona Inkielewicz-Stepniak

**Affiliations:** 1 Department of Pharmaceutical Pathophysiology, Medical University of Gdansk, 80-210 Gdansk, Poland; 2 Department of Molecular Biochemistry, University of Gdansk, 80-309 Gdansk, Poland; 3 Bioimaging Laboratory, Faculty of Biology, University of Gdansk, 80-309 Gdansk, Poland; 4 Department of General Endocrine and Transplant Surgery, Medical University of Gdansk, 80-210 Gdansk, Poland; 5 Department of Chemistry, University of Ioannina, 45110 Ioannina, Greece; 6 University Research Center of Ioannina, Institute of Materials Science and Computing, 45110 Ioannina, Greece

**Keywords:** opioid growth factor, [Met^5^]-enkephalin, gemcitabine, peptide-drug conjugates, pancreatic cancer, pancreatic cancer organoids, selective cytotoxicity, platelets, TCIPA

## Abstract

Pancreatic ductal adenocarcinoma (PDAC) presents a formidable challenge with high lethality and limited effective drug treatments. Its heightened metastatic potential further complicates the prognosis. Owing to the significant toxicity of current chemotherapeutics, compounds like [Met^5^]-enkephalin, known as opioid growth factor (OGF), have emerged in oncology clinical trials. OGF, an endogenous peptide interacting with the OGF receptor (OGFr), plays a crucial role in inhibiting cell proliferation across various cancer types. This in vitro study explores the potential anticancer efficacy of a newly synthesized OGF bioconjugate in synergy with the classic chemotherapeutic agent, gemcitabine (OGF-Gem). The study delves into assessing the impact of the OGF-Gem conjugate on cell proliferation inhibition, cell cycle regulation, the induction of cellular senescence, and apoptosis. Furthermore, the antimetastatic potential of the OGF-Gem conjugate was demonstrated through evaluations using blood platelets and AsPC-1 cells with a light aggregometer. In summary, this article demonstrates the cytotoxic impact of the innovative OGF-Gem conjugate on pancreatic cancer cells in both 2D and 3D models. We highlight the potential of both the OGF-Gem conjugate and OGF alone in effectively inhibiting the ex vivo pancreatic tumor cell-induced platelet aggregation (TCIPA) process, a phenomenon not observed with Gem alone. Furthermore, the confirmed hemocompatibility of OGF-Gem with platelets reinforces its promising potential. We anticipate that this conjugation strategy will open avenues for the development of potent anticancer agents.

## 1. Introduction

Pancreatic cancer has one of the worst prognoses of all cancers. According to GLBOCAN statistics, in 2020, 495,773 people developed pancreatic cancer, and 466,003 died of the disease [1]. The high mortality rate, 94%, is mainly due to the cancer’s increased resistance to treatment and late-stage disease diagnoses. In most cases, pancreatic cancer does not show any specific symptoms in the initial stage of cancer development. Symptoms that may indicate pancreatic cancer include abdominal pain, weight loss, lethargy, depression, nausea, vomiting, changes in bowel movements, back and shoulder pain, pruritus, and jaundice [2,3]. In 90% of cases, pancreatic oncogenesis occurs around the age of 40–55. The incidence of pancreatic cancer in men and women increases with age. Risk factors include smoking, chronic hepatitis, high-fat diets, excessive alcohol consumption, physical inactivity, obesity, and diabetes [2]. Individuals with diabetes, particularly type 2, might face a higher susceptibility to the onset of pancreatic cancer in comparison to those without diabetes. It is crucial to underscore that pancreatic cancer is a multifaceted condition, and diabetes represents just one among several significant risk factors [3,4,5]. Depending on the tumor’s advancement, patients undergo tumor resection and either chemotherapy or radiotherapy. Unfortunately, only 10–20% of patients with advanced cancer are eligible for surgery. Patients in advanced stages of pancreatic cancer are treated only with classical chemotherapeutic drugs and radiotherapy. Specifically, gemcitabine (Gem); 5-fluorouracil, folinic acid, oxaliplatin, and irinotecan (FOLFIRINOX); and albumin-bound paclitaxel (nab-paclitaxel) are commonly used as therapeutic schemes [6]. However, Gem, the most commonly utilized drug, carries the risk of rapid inactivation in the body (it produces the unwanted metabolite 2′,2′-difluorodeoxyuridine; dFdU) and is also highly toxic to non-cancerous tissues. To overcome this hurdle, conjugates containing Gem have been developed and shown to improve in vitro activity against various cancer cells. Such a peptide–drug conjugation is considered a viable and promising approach for improving drug targeting and enhancing drug pharmacokinetic profiles. For instance, the Gem-linker-GnRH conjugates have reportedly improved drug efficiency by enhancing solid-tumor targeting [7,8,9,10].

[Met^5^]-enkephalin, or opioid growth factor (OGF), is an endogenous pentapeptide. OGF forms a biological axis with the OGF receptor (OGFr) located on the nucleus membrane [11]. Several studies have confirmed in vitro and in vivo tumor cell proliferation inhibition by OGF in human colon tumors, squamous cell carcinoma of the head and neck, and kidney, ovarian, and pancreatic cancer [12]. Proliferation inhibition in pancreatic cancer has been tested against four commercially available pancreatic cancer cell lines (Mia PaCa-2, Capan-1, BxPC-3, and PANC-1). Pancreatic cancer cells are arrested in the G_1_/S phase of the cell cycle [13]. Notably, extensive OGF research has led to the approval of the pentapeptide in phase I and II clinical trials. OGF therapy is more effective when combined with standard chemotherapy treatments, such as Gem and 5-fluorouracil. Combination therapy is associated with increased tumor growth inhibition by suppressing DNA synthesis. OGF also inhibits cell migration, prolongs diagnosed patients’ lives, and reduces chemotherapy side effects in patients with inoperable pancreatic cancer [11,14,15].

The important problem associated with various types of cancer, including pancreatic cancer, is metastasis. Interactions between Circulating Tumor Cells (CTCs) and platelets—TCIPA play a significant role in cancer progression [16,17,18]. The basis of the platelet–cancer cell interaction is the binding of surface receptors, GP IIb/IIIa, and P-selectin. The coating of cancer cells by platelets serves as a physical barrier to protect cancer cells from immune destruction. Moreover, cancer cells cause the release of various factors from the granules such as TGF-β, impairing the mobilization and cytolytic activity of Natural Killer (NK) cells. Also, factors that stimulate the growth of tumor tissue and angiogenesis are released from the platelet granules. ADP causes the loosening of connections between cells by interacting with the receptors of the blood vessel epithelium, which facilitates the migration of cancer cells. Platelets also release metalloproteins, which contribute to the digestion of the extracellular matrix and create a new environment for the tumor. Thus, the development of effective drugs that inhibit the TICPA process would help to reduce the pancreatic cancer progression [16,17,18,19,20,21].

**Figure 1:**
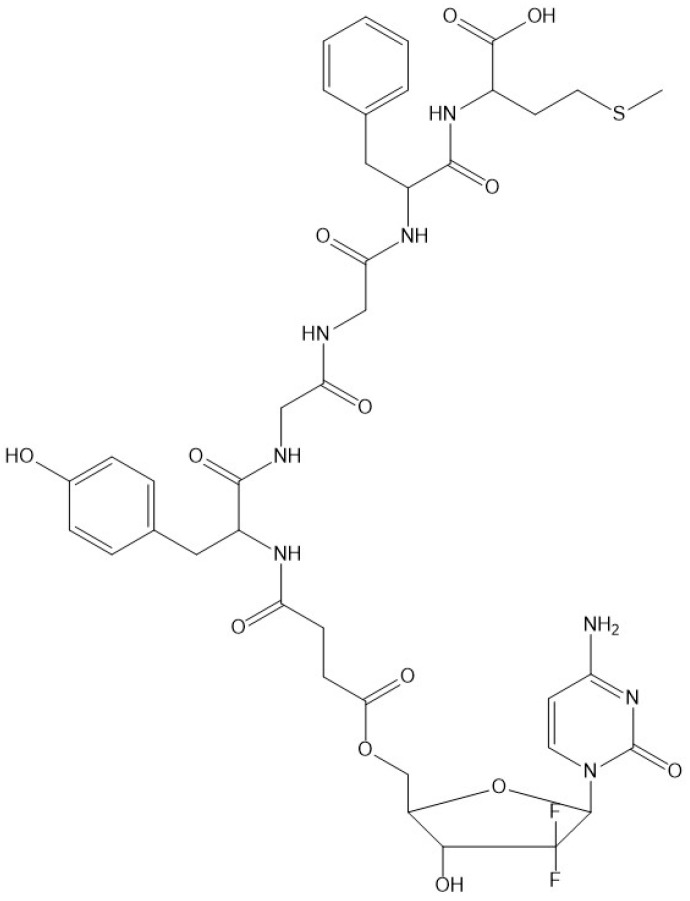
Structure of the OGF-Gem conjugate.

Therefore, we designed, synthesized, and characterized an OGF-Gem conjugate, where OGF and Gem are tethered by an organic linker (Figure 1). Gem was subjected to selective protection using the tert-butoxycarbonyl (Boc) group and prepared as gemcitabine hemisuccinate. 5′-O-diBoc-gemcitabine hemisuccinate was conjugated with the OGF peptide in solution. We demonstrated the cytotoxic activity of the OGF-Gem conjugate against pancreatic cancer cell lines, including the metastatic line (MIA PaCa-2 and AsPC-1). Furthermore, we confirmed that OGF-Gem is either not cytotoxic or significantly less cytotoxic to two non-tumor-transformed human cells—kidney (HEK-293) and skin fibroblast cells (HDFa). We also determined the effect of OGF-Gem on cell cycle inhibition, and the inhibition of cell proliferation, senescent cells, and apoptosis. We have demonstrated that OGF-Gem has antimetastatic potential due to inhibited pancreatic tumor cell (AsPC-1)-induced platelet aggregation. This can significantly impact the inhibition of disease progression (metastasis) of pancreatic cancer.

## 2. Materials and Methods

### 2.1. Selective Boc Protection of Gem

Gem was selectively Boc-protected to obtain compound **1**, as previously presented [7,8]. Then, the diBoc-protected Gem (1) was reacted with succinic anhydride under basic conditions to afford the 5′-O-diBoc-gemcitabine hemisuccinate (2) [22]. In short, the diBoc-protected Gem (1) (0.43 mmol) and succinic anhydride (1.08 mmol) were dissolved in 20 mL of anhydrous dichloromethane (CH_2_Cl_2_). Then, DIPEA (4.30 mmol) was added dropwise to this solution. The resulting mixture was stirred at room temperature for 4 h (reaction monitored via TLC in solvent system, CH_2_Cl_2_/methanol 9/1, R_f_ = 0.04), and then, 10 mL of water was added, and the mixture was lyophilized. The crude product was purified using RP-HPLC (60:40% 0.1% TFA in H_2_O: 0.1% TFA in acetonitrile, to 100% 0.1% TFA in acetonitrile for 30 min, at 254 nm, t_R_ = 11.75 min) to afford compound **2** (78%) as a white solid.

### 2.2. Peptide Synthesis

The chemical synthesis of the OGF peptide (Tyr-Gly-Gly-Phe-Met) was carried out automatically (Prelude peptide synthesizer, Gyros Protein Technology, Tucson, AZ, USA) on solid support (2-chlorotrityl chloride resin) at room temperature using the classic Fmoc/tBu strategy. The C-terminal Fmoc-Met-OH was attached to the resin in the presence of an equimolar amount of *N*,*N*-diisopropylethylamine (DIPEA) in anhydrous CH_2_Cl_2_. The loading of the first amino acid was determined spectroscopically by measuring the absorbance of the dibenzofulvene-piperidine adduct at 301 nm (the molar absorption coefficient in ε = 6402 M^−1^ × cm^−1^).

The peptide chain was elongated in consecutive deprotection and coupling cycles. Deprotection was performed with 20% piperidine in *N*,*N*-dimethylformamide (DMF), and peptide chain elongation was achieved using a three-fold molar excess of TBTU/HOBt/NMM and each of the N-α-Fmoc-protected amino acids. After completing the synthesis, the peptide was cleaved from the resin using a mixture of trifluoroacetic acid (TFA)/triisopropylsilane (TIS)/phenol/water (88:5:2:5, *v*/*v*/*v*/*v*). The crude peptides were purified using a PLC 2050 Gilson HPLC (Gilson, France), equipped with a Grace Vydac C18 (218 TP) HPLC column (22 × 250 mm, 10 μm, 300 Å). The solvent system was 0.1% TFA in water (phase A) and 80% MeCN in A (phase B). A linear gradient of 10–43% B was applied for 25 min (flow rate: 20 mL min^−1^, monitored at 226 nm). Synthesis was performed according to a protocol previously described by us [23,24].

### 2.3. Conjugate Synthesis

To a solution of 5′-O-diBoc-gemcitabine hemisuccinate 2 (0.087 mmol), HATU (0.087 mmol), and DIPEA (0.361 mmol) in 12 mL of anhydrous DMF, a solution of the pure OGF peptide (0.079 mmol) in 4 mL of anhydrous DMF was added dropwise. The reaction mixture was stirred at room temperature for 27 h, and the solvent was removed under reduced pressure to produce the crude Boc-protected conjugate. To remove the Boc protective groups, the conjugate was treated with 5 mL of a solution of TFA/H2O/TIS (95:2.5:2.5). The resulting mixture was stirred at room temperature for 4 h and then concentrated under reduced pressure to produce the crude product, which was subsequently purified via HPLC using a PLC 2050 Gilson HPLC (Gilson, Saint-Avé, France), equipped with a Grace Vydac C18 (218 TP) HPLC column (22 × 250 mm, 10 μm, 300 Å). A linear gradient of 15–55% B was applied for 35 min (flow rate: 20 mL min^−1^, monitored at 226 nm). Conjugate integrity and stability were checked via RP-HPLC on a Shimadzu Prominence UFLC equipped with a Phenomenex, Kinetex 5u, XB-C18 100Å 150 × 4.6 mm, and a UV-Vis detector. The solvent system was 0.1% TFA in water (A) and 80% MeCN in A (B). A linear gradient from 10% to 90% B within 30 min., with a flow rate of 1.0 mL/min and monitoring set at 226 nm, was employed. A mass spectrometry analysis was carried out on a MALDI MS (Bruker AutoFlex Max MALDI-TOF Mass Spectrometer) using an α-cyano-4-hydroxycinnamic acid (CCA) and/or 2,5-dihydroxybenzoic acid (DHB) matrix. Synthesis was performed according to a protocol previously described by us [23,24].

### 2.4. Stability of OGF-Gem Conjugate and OGF

The OGF-Gem conjugate and OGF alone were incubated in a medium (DMEM) supplemented with 10% FBS at a temperature of 37 °C. The initial concentrations of the peptide and conjugate solutions were both 0.5 mM. Samples of 60 μL were taken immediately after the start of incubation, 30 min, 90 min, 180 min, and 23 h and mixed with 60 μL of acetonitrile to separate the proteins. The formed precipitate was removed via centrifugation for 15 min at 13,000 rpm. The supernatant was analyzed via RP-HPLC [(Shimadzu Prominence, Duisburg, Germany) linear gradient 10–90% phase B, 20 min., column Kinetex 5 μm XB-C18 100Å 150 × 4.6 mm] and MALDI-MS (Autoflex maX MALDI-TOF spectrometer, Bruker Daltonics, Bremen, Germany) using a 2,5-dihydroxybenzoic acid matrix (DHB). A stability analysis was performed according to a protocol previously described by us [23,24].

### 2.5. Cell Lines and Cell Cultures

The MIA PaCa-2 and AsPC-1 pancreatic cancer cell lines were obtained from the American Type Culture Collection (ATCC; Manassas, VA, USA). MIA PaCa-2, a human pancreatic cancer cell line, was isolated from the pancreatic duct (ATCC, CRL-1420) and cultured in Dulbecco’s Modified Eagle’s Medium (DMEM), supplemented with 10% FBS, 2.5% horse serum, and 1% penicillin/streptomycin (P/S). AsPC-1, a human metastatic pancreatic tumor cell line (ATCC, CRL-1682), was cultured in Roswell Park Memorial Institute 1640 Medium, supplemented with 10% FBS and 1% P/S. HDFa, a human primary dermal fibroblast was obtained from the American Type Culture Collection (ATCC; Manassas, VA, USA). The HDFa was cultured in DMEM and supplemented with 10% FBS and 1% P/S. HEK-293 immortalized human embryonic kidney was obtained courtesy of Professor Ewa Augustin from the Gdansk University of Technology. The HEK-293 was cultured in DMEM, and supplemented with 10% FBS and 1% P/S. Cells were cultured at 37 °C in a humidified atmosphere with 5% CO_2_. The cells were maintained in a 75 cm^2^ tissue culture flask. The medium was replaced every two days. The subcultivation ratios were 1:3 for AsPC-1 and HDFa cells, 1:4 for MIA PaCa-2 cells, and 1:6 for HEK-293 [25,26,27]. 

Pancreatic cancer tissues were obtained from patients undergoing surgical resection at the university center clinic of the Medical University of Gdansk (GUMed, Gdansk, Poland). Organoids from patients 3 and 4 were developed at the Department of Pharmaceutical Pathophysiology of Medical University of Gdansk, while pan-80 organoids were kindly offered by CELLphenomics GmbH (Berlin, Germany). pan-87 organoids were kindly developed at Dr. Vincenzo Corbo’s lab (University of Verona, Italy) and obtained from CELLphenomics GmbH. All human experiments were approved by the ethics committee. Written informed consent for the research-based use of tissue in this study was obtained from the donors prior to specimen acquisition. Samples were confirmed to be tumor or non-tumor-transformed based on pathological assessment. Tumor tissue was minced and digested with collagenase II (5 mg/mL), Dispase I (1.25 mg/mL), and Y-27632 Dihydrochloride (10.5 μM) in human HSM^+++^ medium at 37 °C and shaken for a maximum of 2 h. The material was further digested with TrypLE Express with DNAse I (0.1 mg/mL) for 10 min at 37 °C, washed with HSM^+++^ medium, embedded in growth factor reduced Matrigel (Corning, #356231, New York, NY, USA), and cultured in human complete medium: HSM^+++^ (AdDMEM/F12 medium supplemented with HEPES, Glutamax, Primocin (0.1 mg/mL)), Wnt3a-conditioned medium (50% *v*/*v*), R-Spondin1-conditioned medium (10% *v*/*v*), B27, N-acetyl-L-cysteine (1.25 mM), mNoggin (100 ng/mL), EGF (50 ng/mL), hGastrin (10 nM), fibroblast growth factor 10 (FGF10, 100 ng/mL), Nicotinamide (10 mM), A83-01 (0.5 μM), and Y-27632 Dihydrochloride (10.5 μM) [28].

### 2.6. MTT Assay

In a 96-well plate, 7 × 10^3^ MIA PaCa-2 and AsPC-1 cells and 3 × 10^3^ HEK-293 and 10^4^ HDFa cells were seeded per well. After 24 h of preincubation in standard conditions, the cells were exposed to the OGF-Gem conjugate (1.56–100 nM), OGF (100 nM), and Gem (100 nM) for 72 h. Cell viability was assessed using the MTT assay. After incubation, the media was supplemented with the MTT assay [water-soluble 3-(4,5-dimethylthiazol-2-yl)-2,5-diphenyltetrazolium bromide, at a final concentration of 0.5 mg/mL]. The plates were incubated at 37 °C in 5% CO_2_ for 2 h. After incubation, the media were aspirated, and formazan crystals were diluted in 100 μL of dimethyl sulfoxide. Next, cell viability was assessed by measuring absorbance at 570 nm with a reference filter of 660 nm using a microplate reader. Cell viability was determined as a percentage of the control (the control cell viability was set at 100%) [25,26,27].

### 2.7. Organoid 3D Viability Assay

The organoids were maintained in Matrigel with human complete medium. The Matrigel was dissociated using a Cell Recovery Solution. The organoids were dissociated into single cells using TrypLE Express with DNAse I (0.01 mg/mL). Cells were counted and diluted to 100 cells/μL in GFR Matrigel with a mixture of complete media and Y-27632 Dihydrochloride. In 96-well plates, 100 μL of this mixture and 10 μL Matrigel with cells (1000 cells/well) were added. After 72 h of incubation, the organoids were treated with gradient concentrations of OGF-Gem prepared in HSM^+++^ medium. Cell viability was assessed after 72 h of incubation using Cell-Titer Glo 3D reagent according to the manufacturer’s instructions. Luminescence was measured on a plate reader [28]. 

### 2.8. BrdU Assay

In a 96-well plate, 7 × 10^3^ MIA PaCa-2 and AsPC-1cells were seeded per well. After 24 h of preincubation in standard conditions, the MIA PaCa-2 and AsPC-1 cells were treated with the compound described in the MTT assay section. Cell proliferation was assessed using the BrdU assay (ELISA assay) according to the protocol after 72 h of incubation. The cells were incubated with BrdU solution for 3 h. Next, they were fixed for 30 min using FixDenat solution and then incubated with an anti-BrdU conjugate antibody for 1.5 h at 21 °C. The cells were rinsed 3 times. Next, a substrate solution was added. After 5 min, the reaction was stopped with a solution of 1 M H_2_SO_4_. Cell proliferation was assessed by measuring absorbance at 490 nm with a reference filter of 690 nm using a microplate reader. Proliferation was determined as a percentage of the control (control cell proliferation was set at 100%) [26].

### 2.9. Cell Cycle Analysis

In a 6-well plate, 1.7 × 10^5^ MIA PaCa-2 and AsPC-1 cells were seeded per well. Briefly, after 24 h of preincubation in standard conditions, the MIA PaCa-2 and AsPC-1 cells were treated with the compound described in the MTT assay section and incubated for 72 h. Both the floating and attached cells were collected and washed with ice-cold PBS. The cells were fixed overnight with 70% ethanol at 4 °C. The ethanol was removed via centrifugation, and the cells were resuspended in PBS containing RNAse A (50 μg/mL) and propidium iodide (50 μg/mL). After a 25 min incubation at 37 °C, the cells were analyzed via flow cytometry. Debris and droplets were removed by gating the appropriate population on FSC/SSC and FL_2_-A/FL_2_-W plots before analysis. The number of cells in each cell cycle phase was determined using dedicated software 6.0 Cell Quest Pro (Becton Dickinson, Franklin Lakes, NJ, USA) (15,000 counts were measured per sample) [26].

### 2.10. Cell Senescence and Flow Cytometry

In a 6-well plate, 1.7 × 10^5^ MIA PaCa-2 and AsPC-1 cells were seeded per well. After 24 h of preincubation in standard conditions, the cells were exposed to the OGF-Gem conjugate (3.125, 12.5, 50, and 100 nM), OGF (100 nM), and Gem (100 nM; 1000 nM—positive control) for 72 h. After incubation, the floating and attached cells were collected using a 1% BSA solution and fixed with 2% paraformaldehyde in PBS for 10 min at room temperature. The cells were washed with 1% BSA solution and incubated with the Senescence Detection Kit for 1.5 h at 37 °C without CO_2_, protected from light. Next, cell senescence was measured via flow cytometry (λ_ex_ 490 nm; λ_em_ 514 nm). Ten thousand events were measured per sample. The data were expressed as a percentage of untreated cells (set to 100%), which were the control [29].

### 2.11. Cell Senescence and Optical Microscopy

In a 12-well plate, 10^5^ PaCa-2 and AsPC-1 cells were seeded per well. After 24 h of preincubation in standard conditions, the cells were exposed to the OGF-Gem conjugate (3.125, 12.5, 50, and 100 nM), OGF (100 nM), and Gem (100 nM, 1000 nM-positive control) for 72 h. The medium was removed from each well and washed with a PBS (pH 7.2) buffer. The cells were incubated with fixative solution (0.2% glutaraldehyde, 2% paraformaldehyde, and a PBS buffer) for 5 min at room temperature. The cells were washed with a PBS (pH 6.0) buffer. Next, the cells were incubated with a staining solution (1 mg/mL X-Gal/DMF, 5 mM K_3_Fe(CN)_6_, 5 mM K_4_Fe(CN)_6_, 2 mM MgCl_2_, 150 mM NaCl, and 40 mM citric acid/buffer Na_2_HPO_4_) for 16 h at 37 °C, without CO_2_. After incubation, the cells were observed using an optical microscope (magnification 20x) [30].

### 2.12. Preparation of Platelets from Whole-Blood and Pancreatic Cancer Cells

Blood was collected from healthy volunteers who had not taken any drugs affecting platelet activation for at least 14 days prior to the experiment. Washed platelets at physiological concentration (250,000/μL) were prepared according to the protocol developed by Radomski and Moncada, 1983. Before each blood collection, volunteers provided their informed consent, the documentation of which is archived at the Department of Pharmaceutical Pathophysiology, Medical University of Gdansk (MUG). Approval for this study was obtained from the MUG Ethics Committee (NKBBN/676-173/2022.). 

The MIA PaCa-2 and AsPC-1 cell cultures were washed and detached with 7 mM EDTA buffer. Next, the buffer was aspirated, and cells were centrifuged for 5 min at 1200 rpm and resuspended in Tyrod buffer. For TCIPA determination, MIA PaCa-2 cells were prepared at different concentrations: 10^5^, 2 × 10^5^, 5 × 10^5^, 10^6^ cells/mL; and AsPC-1 cells at concentrations of 5 × 10^4^, 10^5^, 2 × 10^5^ cells/mL [31,32,33].

### 2.13. The Effect of the OGF-Gem Conjugate on TCIPA

The interactions between platelets and pancreatic tumor cells were measured via transmission light aggregometry. In total, 250,000/μL WP samples were placed in a channel of aggregometer (Chronolog, Havertown, PA, USA) and incubated for 2 min, 37 °C, with stirring at 900 rpm to exclude the spontaneous aggregation of thrombocytes. Afterward, MIA PaCa-2 and AsPC-1 cells (at the concentrations indicated in Section 2.12) or collagen (2 μg/mL used as a control agonist) were added, and platelet aggregation was analyzed using the Aggro-Link data-processing system (Chronolog, Havertown, PA, USA) for 30 min. To assess the effect of the tested compounds on the TCIPA, OGF-Gem conjugate, and OGF at the final concentrations of 10, 50, and 100 μM or Gem at 50, 100 μM were added to the WPs and incubated for 10 min, and then AsPC-1 cells (2 × 10^5^/mL) were added and analyzed within 30 min. Platelet aggregation was expressed as the percentage of aggregation at 45 min of the experiment [31,32,33].

Samples collected after the TCIPA determination using the light aggregometer were fixed in 2.5% glutaraldehyde. The samples were then stained with 1% OsO_4_, gradually dehydrated with ethanol solution (30–100%), and embedded in EPON solution. Slides were prepared using a Leica UC7 ultramicrotome and stained with Uranyless and Reynolds lead citrate. Preparations were analyzed using a Tecnai G_2_ Spirit BioTWIN TEM [31].

### 2.14. LDH Release Assay

The isolated WP (prepared as described in Section 2.12) were transferred to a 96-well plate for fluorescence measurements. Next, the OGF-Gm conjugate and the OGF at 50 and 100 μM concentrations were added. The control sample consisted of platelets suspended in Tyrod buffer. The positive control was WP treated with lysis buffer (0.1% Triton X-100) and the lysis buffered-WPs were set to 100%. Platelets with tested compounds were incubated for 15 min at 37 °C. Then, 100 μL of the LDH assay was added to each well and incubated for another 10 min, protected from light. The reaction was stopped with a Stop Solution buffer. Fluorescence intensity was measured with a plate reader (λ_ex_ 560 nm; λ_em_ 590 nm). The results were expressed as the percentage of the total LDH release [31].

### 2.15. Statistical Analysis

Data were analyzed using the GraphPad Prism 5 software and presented as the mean ± SD of 3–4 independent experiments. The statistical analysis was determined through one-way ANOVA and Tukey’s post hoc test.

## 3. Results

### 3.1. Selective Boc Protection of Gemcitabine

**Figure 2:**
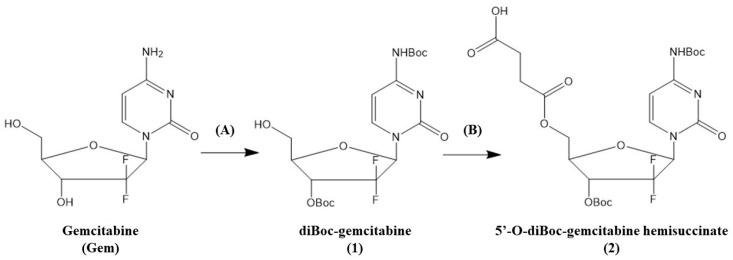
Synthetic schemes. Reagents and conditions: (**A**) (1) Boc_2_O, Na_2_CO_3_, and dioxane-H_2_O (80:20), room temperature (rt), 48 h, (2) Boc_2_O, dioxane, 37 °C, 72 h; (**B**) succinic anhydride, DIPEA, CH_2_Cl_2_, rt, 4 h.

The secondary 3′-OH and 4-NH_2_ moieties of Gem were selectively protected with tert-butoxycarbonyl group (Boc) based on the method developed by Guo and Gallo [17] while a hemisuccinate linker was attached to the primary 5′-OH group (Figure 2), as was described previously by us [7,8,9,10]. Supplementary Figure S1A shows that the homogeneity of the product was confirmed via high-performance liquid chromatography (HPLC). In addition, the structure of the obtained diBoc-protected compound **2** was confirmed through ^1^H and ^13^C NMR analyses (Supplementary Figure S1B,C). The OGF peptide was synthesized on the solid support using the Fmoc/tBu strategy. The homogeneity of the product was confirmed via HPLC (Supplementary Figure S1D). The purified compound **2** was attached to the OGF peptide in solution.

### 3.2. Synthesis and Stability Evaluation of the Conjugate Compounds

In the last step, both Boc protecting groups were removed with a mixture of trifluoracetic acid (TFA), water, and triisopropylsilane (TIS). Supplementary Figure S1E shows that the homogeneity of the final conjugate was analyzed via HPLC. The identity of the conjugate was confirmed via MALDI-TOF (Supplementary Figure S1F). The stability of the OGF peptide and its conjugate, OGF-Gem, were examined in medium (DMEM) supplemented with 10% FBS. Intact OGF was still present after 23 h of incubation (Supplementary Figure S4F); however, as shown through the HPLC analysis, its concentration was lower as compared to the initial state (Supplementary Figure S4B). A closer analysis of the MS data revealed the presence of *m*/*z* signals with low intensities that may correspond to the truncated parent peptide deprived of N-terminal Tyr reside (411.13 [M+H]^+^; 433.10 [M+Na]^+^; 449.08 [M+K]^+^). This may result from enzymatic degradation driven by proteases (an aminopeptidase) found in the medium supplemented with FBS. The presence of intact OGF-Gem conjugate upon incubation with supplemented medium for 23 h (Supplementary Figure S2) and 3 days (Supplementary Figure S3) was confirmed through an HPLC analysis. Supplementary Figure S1F shows a split peak with retention times (11.12 min and 11.26 min) which corresponds well with a double signal recorded for OGF-Gem just after the start of incubation (11.19 min and 11.30 min, Supplementary Figure S2B). The total area of the former signal is smaller and accounts for about 39% of the entire area of the latter peak. This result indicates a decrease in the concentration of the intact OGF-Gem upon incubation. The presence of OGF-Gem after 23 h of incubation was not confirmed through MS analysis. Nevertheless, the most significant observation is the gradual release of free Gem during the incubation period. As shown in Supplementary Figure S3D, the HPLC signal with a retention time of 12.11 min, recorded upon 3 days of incubation in medium, corresponds to the one for Gem alone (Supplementary Figure S3B; t_R_ = 11.54 min). The existence of free Gem and the remaining OGF peptide with the attached succinyl linker was also confirmed through MS analysis (Supplementary Figure S2D–F). Upon 23 h of incubation, the *m*/*z* signal 696.18 corresponding to the [M+Na]^+^ of OGF with succinate linker is the most intensive one. The ester bond connecting the 5′-OH group of Gem and the carboxyl group of the succinyl linker attached to the OGF peptide is known to be prone to hydrolysis, which leads to the release of the parent drug. It is worth noting that in the case of both compounds tested, OGF and OGF-Gem, the *m*/*z* signals corresponding to their analogues with oxidized Met residue were also recorded through MS analysis (Supplementary Figures S2 and S4). In our case, however, the recorded signals for compounds bearing Met(O) had very low intensities as compared to their counterparts with non-oxidized Met residue.

### 3.3. Assessment of the OGF-Gem Conjugate’s Impact on Cell Viability in a 2D Model and Its Effect on Pancreatic Cancer Organoids in a 3D Model

**Figure 3:**
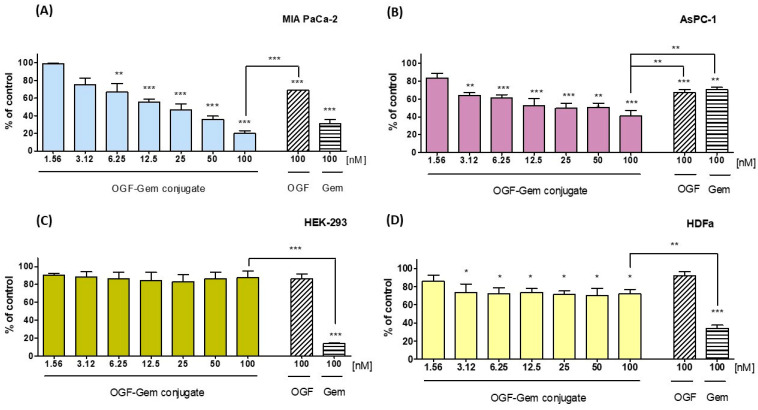
The effects of the OGF-Gem conjugate on the viability of pancreatic cancer cell lines: (**A**) MIA PaCa-2, (**B**) AsPC-1 and non-tumor transformed cells, (**C**) HEK-293, and (**D**) HDFa, as measured through an MTT assay after 72 h of incubation. Data are the mean ± SD of three separate determinations. * *p* < 0.05 ** *p* < 0.01; *** *p* < 0.001 as compared to the control (untreated) cells and the 100 nM OGF-Gem conjugate.

The tested compound’s cytotoxic activity was determined using the MTT test, which is based on the ability to convert tetrazole salts to water insoluble formazan through mitochondrial dehydrogenases [34]. Our results show a high cytotoxic effect on all pancreatic cancer cell lines (Table 1). Exposing pancreatic cell lines MIA PaCa-2 and AsPC-1 to OGF-Gem decreased viability (Figure 3A,B). Importantly, the OGF-Gem conjugate demonstrated a more pronounced cytotoxic effect against the metastatic pancreatic cancer cell line AsPC-1 compared to the commonly used chemotherapeutic agent. The results obtained for non-tumor-transformed cells—a human embryonic kidney line HEK-293 and human primary dermal fibroblast line HDFa presented a slight cytotoxicity effect from the OGF-Gem derivative within 3 days of incubation for all tested concentrations (Figure 3C,D). Interestingly, an 80% reduction in HEK-293 cell viability was observed for the 100 nM Gem compared to the 100 nM OGF-Gem derivative. In HDFa cells, 100 nM Gem reduced viability to 35%, while the OGF-Gem conjugate slightly decreased the viability (to 75% viability) after 72 h of incubation. Based on the analysis of the results, concentrations of 3.125, 12.5, 50, and 100 nM, as well as an incubation time of 72 h, were selected for further experiments on the three pancreatic cancer cell lines.

**Figure 4:**
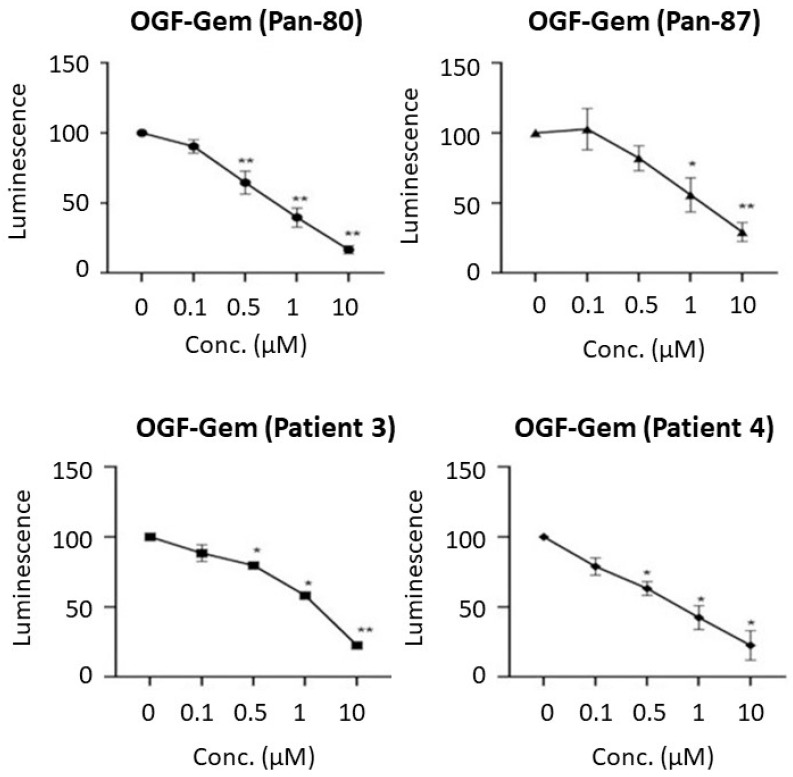
The OGF-Gem conjugate decreased the viability of pancreatic cancer patient organoids, as determined using the CellTiter Glo 3D assay after 72 h incubation. The nomenclatures of the two organoids, namely patient 3 and patient 4, adhere to the regulations stipulated by the biobank at the Medical University of Gdansk, and the other two organoids (Pan-80, Pan-87) are according to the principle of the biobank at CELLphenomics GmbH and University of Verona. Data are mean ± SD of three separate determinations. * *p* < 0.05; ** *p* < 0.01, as compared to the control (untreated) cells.

Figure 4 shows that the pancreatic cancer organoids derived from four patients decreased in viability after 72 h of exposure to the OGF-Gem conjugate. To assess viability, the CellTiter-Glo 3D test was used, involving luminescence readings correlating with the ATP concentration in living cells [35]. As the OGF-Gem conjugate concentration increased (0.1–10 μM), the viability of the pancreatic cancer organoids decreased for all four patients.

### 3.4. The Impact of OGF-Gem on Cell Proliferation and the Cell Cycle

**Figure 5:**
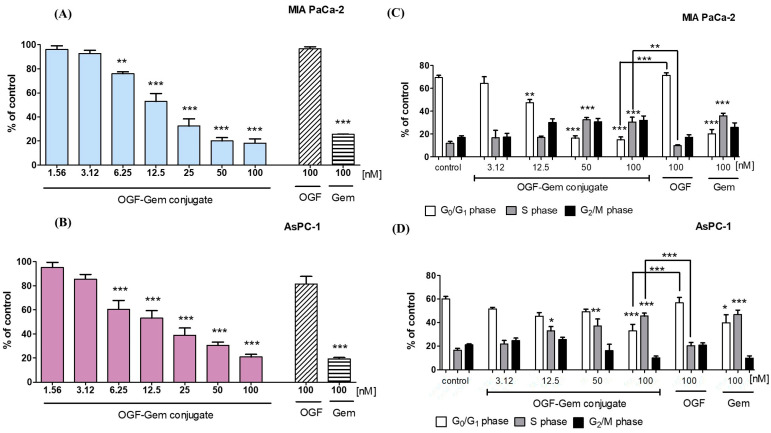
The OGF-Gem conjugate decreased the proliferation of pancreatic cancer cells: (**A**) MIA PaCa-2 and (**B**) AsPC-1. Proliferation was measured using the BrdU assay after 72 h of incubation. The effect of the OGF-Gem conjugate on the cell cycle distribution for (**C**) MIA PaCa-2 and (**D**) AsPC-1 cells after 72 h of incubation. Data are mean ± SD of three separate determinations. * *p* < 0.05; ** *p* < 0.01; *** *p* < 0.001, as compared to the control (untreated) cells and 100 nM of the OGF-Gem conjugate.

The study delved into examining the impact of the OGF-Gem conjugate on suppressing cancer cell proliferation and influencing the cell distribution across the three phases after a 72 h incubation period. The BrdU test assesses cell proliferation, which involves incorporating bromodeoxyuridine into the DNA of actively proliferating cells [36]. Figure 5A,B illustrate the tested conjugate’s antiproliferative nature against all pancreatic cancer lines. Proliferation inhibition correlates with increased conjugate concentrations. The highest concentration tested, 100 nM of the OGF-Gem derivative, reduced cell proliferation in all pancreatic cancer lines by approximately 75%. The OGF at a concentration of 100 nM did not affect the proliferation of MIA PaCa-2 or AsPC-1 cells, as opposed to the 100 nM Gem.

Furthermore, to assess the impact of OGF-Gem on the cell cycle progression of pancreatic cancer lines, samples underwent flow cytometry analysis to quantify the DNA levels corresponding to the G_0_/G_1_ phases, S phase, and G_2_/M phases (Figure 5C,D). The OGF-Gem conjugate decreased the G_0_/G1 phase gap for MIA PaCa-2 and AsPC-1 cells and caused arrest in the S phase of the cell cycle.

### 3.5. The Impact of OGF-Gem on Cellular Senescence

**Figure 6:**
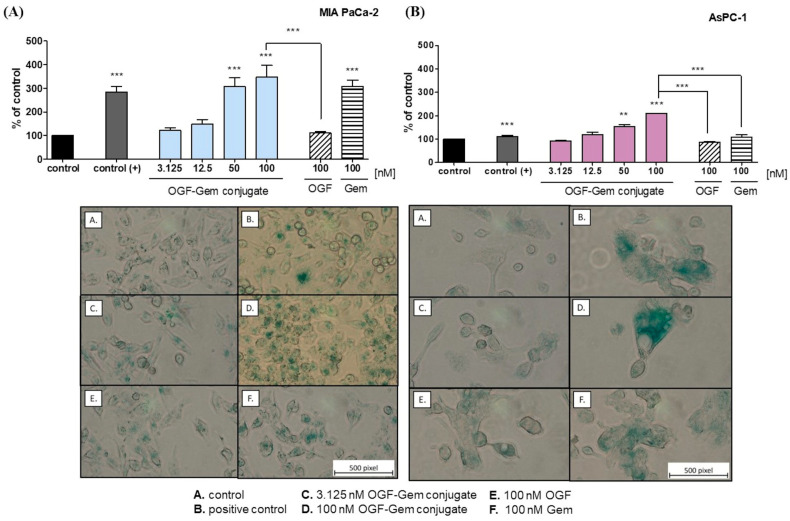
Effects of the OGF-Gem conjugate on induced senescent cells in (**A**) MIA PaCa-2 and (**B**) AsPC-1 cells after 72 h of incubation as measured via flow cytometry and optical microscopy (A. control, B. positive control, C. 3.125 nM OGF-Gem conjugate, D. 100 nM OGF-Gem conjugate, E. 100 nM OGF, and F. 100 nM Gem). Gemcitabine (1 μM) was used as a positive control. Data are mean ± SD of three separate determinations. ** *p* < 0.01; *** *p* < 0.001 as compared with untreated cells and 100 nM of the OGF-Gem conjugate.

Subsequently, the advancement of induced senescent cells was assessed via flow cytometry and optical microscopy. Cell senescence was induced in MIA PaCa-2 cells with increasing concentrations of the OGF-Gem conjugate and 100 nM of Gem (Figure 6A). Stress-induced premature senescence was not caused by 100 nM of OGF in MIA PaCa-2. However, in AsPC-1 cells, senescence occurred only with the OGF-Gem conjugate at 50 and 100 nM (Figure 6B). In AsPC-1 cells, senescence was more significant with the 100 nM OGF-Gem derivative than with the 100 nM OGF and, importantly, than with Gem alone. The optical microscopy results confirm those obtained via flow cytometry (Figure 6). Cells exhibiting accelerated cellular senescence showed significant morphological changes, with a visible blue color following the breakdown of X-Gal by β-galactosidase.

### 3.6. The Impact of OGF-Gem on Cell Apoptosis

**Figure 7:**
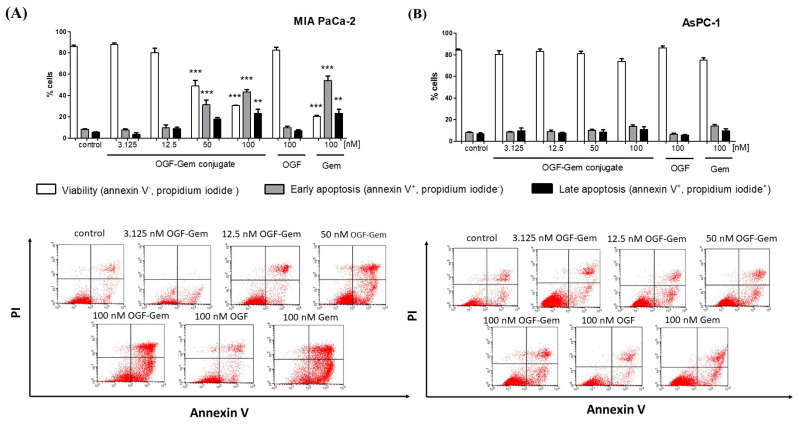
The observed effects of the OGF-Gem conjugate on early and late apoptosis induction in (**A**) MIA PaCa-2 and (**B**) AsPC-1 cells treated for 72 h. The analysis was performed via flow cytometry. Percentage of normal cells (Annexin V^−^/PI^−^), cells in early apoptosis (Annexin V^+^/PI^−^), and cells in late apoptosis (Annexin V^+^/PI^+^). Representative bivariate histograms of Annexin V/PI staining. Data are mean ± SD of three separate determinations. ** *p* < 0.01; *** *p* < 0.001 as compared with untreated cells and 100 nM of the OGF-Gem conjugate.

We then explored the potential of the OGF-Gem conjugate to lead cancer cells to apoptosis. The study substantiated that OGF-Gem prompted apoptosis in MIA PaCa-2 cells (Figure 7A). The findings from MIA PaCa-2 cells indicate that the OGF-Gem conjugate initiates a concentration-dependent programmed cell death process. Concurrently, cell growth was predominantly observed in the early phase of apoptosis, extending into the later stages. No apoptosis was detected in AsPC-1 cells with any of the tested compounds at the examined concentrations (Figure 7B).

### 3.7. The Effect of OGF-Gem Conjugate on TCIPA

**Figure 8:**
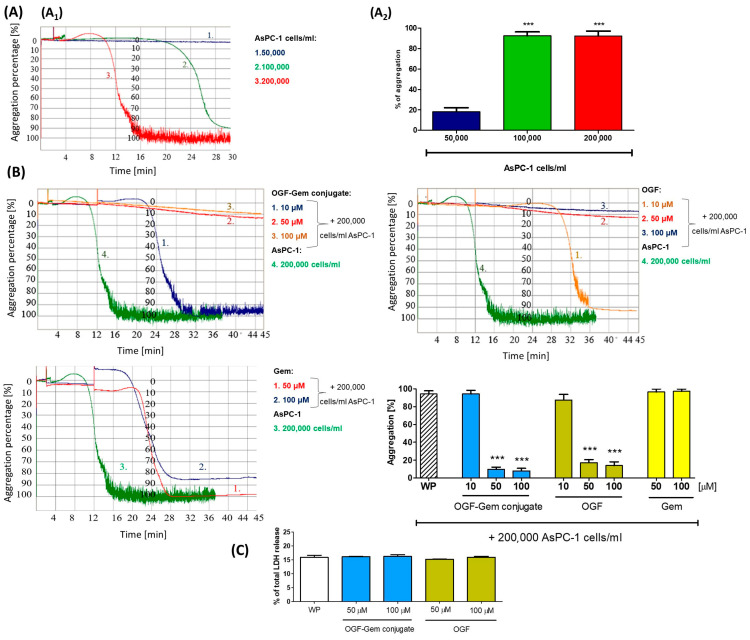
OGF-Gem conjugate inhibits the TCIPA process induced by AsPC-1. (**A**) The induction of platelet aggregation by AsPC-1 pancreatic cancer cells. (**A_1_**) Representative LTA graph showing TCIPA induced by AsPC-1 cells depending on the concentration. (**A_2_**) Data are mean ± SD of five separate determinations. *** *p* < 0.001. (**B**) The OGF-Gem conjugate and OGF (10, 50, 100 μM), but not Gem (50, 100 μM), inhibited TCIPA. Data are mean ± SD from five separate determinations. *** *p* < 0.001 as compared with WP+AsPC-1. (**C**) LDH cytotoxicity assay. The effect of the OGF-Gem conjugate and OGF on the LDH release from WP. Data are the mean ± SD of three separate determinations compared to 100% cytotoxicity (maximum LDH release). Lysis buffer-treated cells and WP were set to 100% (total LDH release).

Our studies show that MIA PaCa-2 cells at concentrations of 100,000; 200,000; 500,000; and 1,000,000 cells/mL did not induce the TCIPA process (Supplementary Figure S5). However, the AsPC-1 cells at concentrations of 100,000 and 200,000 cells/mL induced the TCIPA process (Figure 8A) in a time-dependent manner (Figure 8(A1)). For further testing of the TCIPA process, a concentration of 200,000 cells/mL of the AsPC-1 line was selected. The lack of spontaneous platelet aggregation was confirmed by each experiment. We have shown that the OGF-Gem conjugate, OGF, and Gem do not affect platelet aggregation at the tested concentration. Before each experiment, the lack of spontaneous aggregation was also confirmed. Both the OGF-Gem conjugate and OGF inhibited the TCIPA process already at a concentration of 50 μM (Figure 8B). Gem did not inhibit the TCIPA process at any of the tested concentrations. Also, the hemocompatibility of OGF-Gem and OGF with platelets has been confirmed. As indicated in Figure 8C, the incubation of platelets with 50 and 100 μM of the OGF-Gem conjugate or OGF did not result in significant releases of LDH.

**Figure 9:**
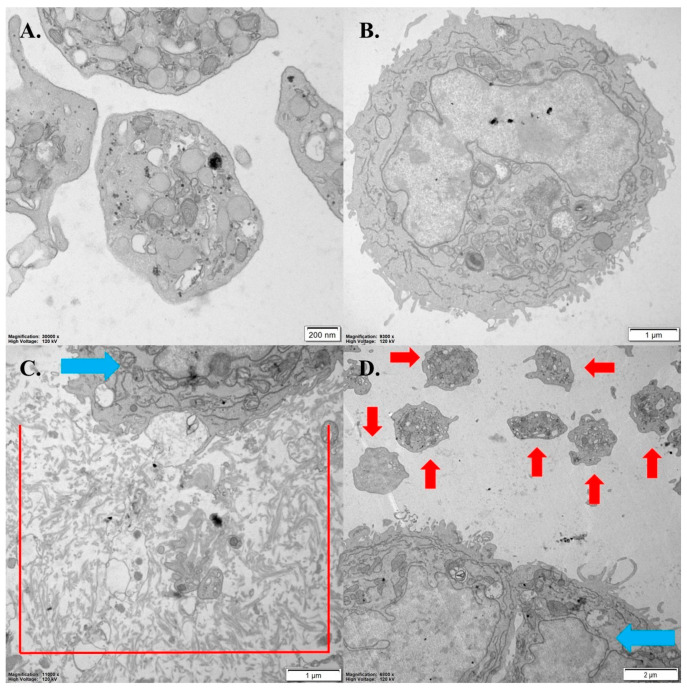
OGF-Gem conjugate inhibits the interaction between platelets and AsPC-1cells (TCIPA). Transmission electron microscopy of (**A**) resting platelets; (**B**) pancreatic cancer cell AsPC-1; (**C**) TCIPA induced by AsPC-1 cells; (**D**) the inhibition of TCIPA in the presence of OGF-Gem conjugate (50 μM). Red arrows indicate platelets; blue arrows, pancreatic cancer cells.

The interaction between platelets and AsPC-1 cells was also observed via TEM (Figure 9). Platelets underwent a change in their morphology, closely encircling the pancreatic cancer cell, forming a platelet–cancer cell aggregate (Figure 9C). In the presence of the OGF-Gem conjugate, the interaction between platelets and pancreatic cancer cells was inhibited (Figure 9D).

## 4. Discussion

**Figure 10:**
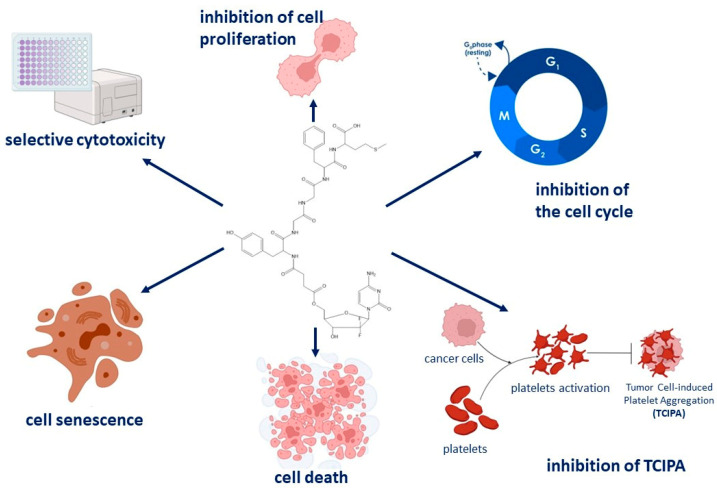
Summary of the assessment of the anti-tumor properties of the new OGF-Gem conjugate on pancreatic cancer cells.

Pancreatic cancer may not be the most prevalent type of cancer, but it holds the lowest survival rate. The efficacy of traditional chemotherapeutic agents is constrained, prompting the search for new chemical compounds for targeted therapy [37]. OGF, an endogenous pentapeptide, binds to the OGFr receptor in pancreatic cancer cells, diminishing the cells’ viability [38]. Currently, research is underway to develop drug-peptide conjugates that will improve pharmacological therapy in pancreatic cancer patients and enhance patient quality of life [39,40]. Zagon et al. demonstrated that combining chemotherapy with Gem and OGF is more effective in inhibiting pancreatic cancer growth in vitro than OGF or Gem alone. They showed that 1000 nM OGF co-administered with 10 nM Gem inhibited PANC-1 and MIA PaCa-2 cell growth [41]. However, no study has compared a pentapeptide to an organic linker-linked chemotherapeutic agent. The findings in this report suggest that the newly synthesized OGF-Gem conjugate effectively diminishes the viability of pancreatic cancer cells, specifically MIA PaCa-2 and AsPC-1. This effect is primarily achieved through the inhibition of proliferation and cell cycle arrest in the S phase. Moreover, the OGF-Gem conjugate demonstrates an induction of cell senescence, with a more pronounced effect observed in AsPC-1 cells compared to exposure to OGF or Gem individually. Furthermore, the OGF-Gem conjugate triggers the apoptotic process in MIA PaCa-1 cells, particularly at higher concentrations. Conversely, no induction of apoptosis was observed in AsPC-1 cells exposed to the OGF-Gem conjugate, suggesting that elevated concentrations may activate alternative forms of cell death, such as autophagy. Additionally, when assessed in a 3D tumor model, the OGF-Gem derivative showcases a reduction in the viability of pancreatic cancer organoids from patients. These comprehensive results provide valuable insights into the potential efficacy of the OGF-Gem conjugate in combating pancreatic cancer (Figure 10).

### 4.1. Effect of OGF-Gem Conjugate on Cell Viability (2D Model) and Pancreatic Cancer Organoids (3D Model)

Our results showed a high cytotoxic effect on all pancreatic cancer cell lines. The selective effect of the OGF-Gem conjugate was evaluated against non-tumor-transformed cells in a human embryonic kidney line, HEK-293, and human primary dermal fibroblasts, HDFa. It has to be emphasized that the HEK-293 and HDFa cell lines are commonly used in research to evaluate the selective cytotoxicity against cancer cells compared to non-cancer cells [42,43,44,45,46,47,48,49,50]. These results demonstrate the selective action of the new OGF-Gem conjugate on pancreatic cancer cells in comparison with classically used chemotherapeutics. The significant cytotoxic effect of Gem is attributed to a higher abundance of nucleoside transporters through which the chemotherapeutic is delivered into normal cells, in contrast to the lower quantity of transporters in pancreatic cancer cells [51]. The OGFr receptor has been identified in many types of cancer cells and normal cells, such as T lymphocytes, keratinocytes, and human umbilical vein endothelial cells. The expression of the OGFr receptor in different cell types varies and depends on many factors. However, the expression of OGFr in pancreatic cancer cells is higher than in normal cells [52,53,54,55,56,57,58]. The above reports may explain the selective effect of our conjugate. Sensitivity to Gem depends on the extent of drug penetration into the cell. Once inside the cell, Gem undergoes di- and triphosphorylation by kinases. The phosphorylated forms of Gem are then incorporated into DNA, simultaneously inhibiting its synthesis [59,60,61]. Gemcitabine is transported across the cell membrane and enters the intracellular space through concentrative (hCNT) and equilibrative (hENT) nucleoside transporters. Once inside the cell, it becomes incorporated into the DNA chain, leading to the inhibition of cell proliferation [62,63,64]. The mechanism by which OGF enters the cell is not fully understood. Additionally, it is possible that OGF may be internalized by the cell via endocytosis [65]. Subsequently, it can bind to the OGFr receptor, which is located on the membrane of the nucleus in cells. Immunoelectron microscopy has shown that OGF co-localizes with OGFr on the nuclear membrane, within the nucleus, and on the periphery of the nuclear heterochromatin. This co-localization suggests that the peptide-receptor complex interacts with caryopherin, facilitating its transport into the nucleus [11,65]. Upon the delivery of OGF to the cell nucleus, CDK4/CDK2 kinase activity decreases. The changes in the cell contribute to the arrest of the cell in the G0/G1 phase of the cell cycle and lead to the inhibition of the proliferation of cancer-transformed cells, which may additionally enhance the effect of gemcitabine [11,66,67]. The site on Gem where OGF is conjugated is an important metabolism/phosphorylation site. The effects of the OGF-Gem conjugate were compared with those of Gem alone at the highest concentration in all the conducted experiments. In the cytotoxicity test for the AsPC-1 line, it was proven that the effect of Gem conjugated with OGF is not weakened and even reduces the viability to a greater extent than the chemotherapeutic agent currently used in medicine, which is Gem. Importantly, we have proven the cytotoxic activity of OGF-Gem also on organoids (3D) model, capable of accurately mimicking the in vivo tumor microenvironment [68,69]. As the OGF-Gem conjugate concentration increased, the viability of the pancreatic cancer organoids decreased for all four patients. As expected, and demonstrated previously, the cytotoxicity was observed at higher concentrations than for the 2D model [69,70]. In addition, stromal cells and immune system cells surround the tumor, effectively promoting tumor growth, drug delivery to the tumor, and tumor resistance. It is possible that this is influenced by the extracellular matrix (ECM). In our research, the ECM consisted mainly of Matrigel. The ECM was a model between patient-derived pancreatic cancer cells and the extracellular matrix (stromal cells) [71,72,73,74,75]. The ECM is a non-cellular three-dimensional macromolecular network composed of collagens, proteoglycans/glycosaminoglycans, elastin, fibronectin, laminins, and several other glycoproteins. Matrigel plays as the same function as ECM in 3D culture, which forms a physical barrier that dissolves or delays drug delivery. Moreover, pancreatic cancer cells’ higher resistance to Gem-OGF in 3D culture organoids compared to the 2D cell model may result from organoid density, the presence of hypoxic zones, increased anti-apoptotic activity, and the interaction of tumor cells with environmental components, the drug ability of penetration, and binding macromolecules—which may explain the use of a higher concentration of the OGF-Gem conjugate in the 3D cell model [76,77,78,79]. These data, from the in vitro 2D model and ex vivo 3D tumor models directly harvested from patients, clearly demonstrate that the OGF-Gem conjugate is effective as a cytotoxic agent against pancreatic cancer. Enhancing the efficacy of pancreatic cancer therapy poses a significant challenge, necessitating the development of novel drug delivery technologies. The stromal microenvironment in pancreatic cancer encompasses crucial cellular components such as activated fibroblasts, mesenchymal progenitor cells, stem cells, transdifferentiated epithelial cells, pericytes, and pluripotent cells derived from adipose tissue. These stromal cells actively contribute to tumor growth and resistance to treatment [80,81]. Szweda et al. demonstrated that polymeric nanocarriers are capable of improving the delivery of OGF to cancer cells [82]. Simultaneously, Greene et al. described how nanocarriers such as metal nanoparticles, polymer nanoparticles, and liposomes can enhance drug delivery to pancreatic cancer cells, despite desmoplasia, and the extensive fibrosis of the stromal framework. Recent studies also indicate that modeling on 3D organoids and humanized mice, reflecting conditions in pancreatic cancer, will contribute to the development of a nanocarrier foundation, facilitating payload delivery to cancer cells [81,82]. As demonstrated by recent research, drug–peptide, drug–polymer, and antibody–drug conjugates, owing to their reduced sizes, have the potential to significantly enhance therapy effectiveness in the context of pancreatic cancer. Additionally, understanding the characteristics of pancreatic cancer and drug delivery systems, such as cell-penetrating peptides (CPPs) and nanocarriers, opens avenues toward reducing therapy toxicity [83,84,85]. Peptides, including OGF, a component of the OGF-Gem conjugate, have the potential to operate within various pH ranges in the tumor microenvironment. Generally, peptides exhibit stability across a broad pH spectrum, but extreme pH levels can influence their structure and activity. Genes that encode surface receptors often display heterogeneous expressions among different patients, posing challenges in the targeted delivery of drugs to tumor cells. Kimbrough et al. demonstrated that pH-dependent probes enhance the delivery of compounds despite the acidic tumor pH. Combining the peptide or OGF-Gem conjugate with a pH-sensitive peptide could improve the delivery of the compound to the tumor despite the acidic environment [86,87,88].

### 4.2. The Influence of OGF-Gem on the Proliferation of Cells and Cell Cycle

Genetic changes in neoplastic cells enable rapid and uncontrolled proliferation outside the context of typical tissue development by acquiring the ability to grow autonomously and survive. Therefore, chemotherapeutic agents are primarily intended to stop the excessive proliferation of tumor cells [78]. Gem is a commonly used chemotherapeutic agent, and its mechanism of action involves its incorporation into the cell’s genetic material, thereby inhibiting DNA replication and proliferation [82]. Furthermore, our results demonstrate effective viability measured by the mitochondrial function when Gem inhibited cell division. AsPC-1 cells, being metastatic cells, exhibit increased resistance to chemotherapy with Gem [89,90]. Our results indicate the tested OGF-Gem conjugate’s antiproliferative nature against two pancreatic cancer lines. Genetic changes in neoplastic cells enable rapid and uncontrolled proliferation outside the context of typical tissue development by acquiring the ability to grow autonomously and survive. Therefore, chemotherapeutic, including gemcitabine, agents are primarily intended to stop the excessive proliferation of tumor cells [90]. The OGF and its receptor, OGFr, constitute the OGF-OGFr axis, which plays a crucial role in regulating cell growth and proliferation [11]. Cheng et al. demonstrated that 1000 nM of OGF inhibits the pronormal cell cycles of human umbilical vein endothelial cells (HUVEC) and human epidermal keratinocytes (NHEKs). The proliferation of HUVEC and NHEK cells was reduced by up to 40% by 1000 nM of OGF [91,92,93]. The new OGF-Gem conjugate also inhibited the proliferation of pancreatic cancer cells. Cancer cells, including those of pancreatic origin, are characterized by numerous mutations in genes responsible for the regulation and proper progression of the cell cycle [94,95,96,97,98,99]. Studies have shown cell cycle inhibition through the OGF-OFGr axis in the G_0_/G_1_ phase of pancreatic cancer, colorectal cancer, and cancer of the neck and head [36,38,60,93]. However, according to Hamed et al., Gem stops pancreatic cancer cells in the S phase of the cell cycle [100]. The OGF-Gem conjugate decreased the G_0_/G_1_ phase gap for MIA PaCa-2 and AsPC-1 cells and caused arrest in the S phase of the cell cycle, where DNA replication occurs [100,101,102,103,104]. Despite this, the OGF-Gem conjugate exerts a cytotoxic effect against the pancreatic cancer cells, which increases the chances of developing new drug–peptide conjugates with antitumor potential.

### 4.3. The Influence of OGF-Gem on Cell Senescence

In senescence cells, there is a characteristic increase in the activity of β-galactosidase activity, which is one of the primary markers of the cell senescence process. Cellular senescence is a stable cell cycle arrest. Cellular senescence is associated with numerous molecular changes and a stable cessation of proliferation. Cellular aging can impede tissue repair and regeneration. The activation of tumor suppressor pathways p53/p21WAF1/CIP1 and p16INK4A/pRB plays a crucial role in regulating senescence [91,92]. Zagon et al. described that the specific target of OGF, acting as an inhibitor of cell proliferation in pancreatic cancer associated with senescence cells, is the p21 CKI pathway. OGF significantly reduced proliferation in HUVEC and NHEK cells. The incubation of HUVEC and NHEK cells with OGF induced the expressions of p16INK4a and p21WAF1/CIP1 proteins. The inhibition of p16INK4a or p21WAF1/CIP1 activation markedly attenuated the inhibitory effect of OGF. The presence of p16INK4a and p21WAF1/CIP1 is essential for the inhibition of cell proliferation through the OGF–OGFr axis, and then the induction of the senescence process [50,91,92,93]. As a result, cells with irreversible DNA damage lose their proliferative potential, and permanent cell cycle arrest, the senescence of cells, and cell death occur [105,106]. Our conjugate caused cell senescence induced in MIA PaCa-2 cells with increasing concentrations of the OGF-Gem conjugate. In AsPC-1 cells, senescence was more significant with the OGF-Gem derivative than with the classically used chemotherapeutic agent.

### 4.4. The Influence of OGF-Gem on Cell Apoptosis

Zagon et al. reported that the OGF compound does not induce apoptosis in pancreatic cancer cells. Gem does not induce apoptosis in MIA PaCa-2 cells. Conversely, Pardo et al. showed Gem-induced autophagy in MIA PaCa-2 and PANC-1 cells. Surprisingly, an autophagy inhibitor (3-methyl adenine) reduces apoptosis in Gem-treated cells, showing that autophagy leads to the apoptotic death of cancer cells [107]. The autophagy and apoptosis processes are often interrelated, which determine a cell’s ultimate fate [108]. However, changes in p53 result in chemotherapy resistance, invasiveness, and apoptosis resistance. Multiple studies have demonstrated increased apoptosis resistance in p53-mutated cancer cells or wild-type p53 cancer cells. MIA PaCa-2 cells have a homozygous p53 missense mutation in exon 7 [109]. AsPC-1 cells contain two mutant p53 alleles and completely lack p53 mRNA transcripts [110]. The MIA PaCa-2 results show that the OGF-Gem conjugate causes a concentration-dependent programmed death process. The decrease in viability that can be observed in all used pancreatic cancer lines is due to the inhibition of the cell cycle. However, the different effects of OGF-Gem conjugate exposure to pancreatic cancer cells probably result from the genotype cells used in the studied [111,112,113,114]. Gerhard et al. demonstrated the expressions of all the components of apoptosis (caspase-3, caspase-9, cytochrome c, Apaf-1) in ten different pancreatic cancer cell lines, including MIA PaCa-2 and AsPC-1. However, differences existed among the cell lines in terms of caspase distribution and cytochrome c-induced activity, which also impact the induction of apoptosis [115,116].

### 4.5. The Impact of OGF-Gem Conjugate on TCIPA

Unfortunately, pancreatic cancer is diagnosed in patients at a late stage, when there are already metastases to other tissues, liver, peritoneum, lungs, and bones. The process of cancer metastasis is also associated with the formation of embolisms in the circulatory system caused by the appearance of aggregates between the CTCs and the platelets [16,19,117,118]. Thrombocytes play a significant role in cancer progression, contributing to the free circulation of CTCs within the blood. In addition, abnormalities in the structure of blood platelets and an increased risk of hypercoagulability are observed in oncological patients. Lim et al. reported that 90.7% of CTCs obtained from pancreatic cancer patients were covered by platelets. Furthermore, the research revealed a positive correlation between the growth of CTC clusters, consisting of platelets, macrophages, and fibroblasts, and the progression of cancer [119]. In addition to playing a pivotal role in the metastatic process, blood platelets are also implicated in the acquisition of drug resistance in pancreatic cancer and the fibrosis process of the disease [120,121,122,123]. The high thrombotic risk in cancer patients is due to the activation and stimulation of platelets to aggregation by CTCs. Cancer cells release mediators such as ADP, TXA2, and thrombin to activate the next platelets. Activated thrombocytes also release amounts of ADP from their granules, promoting subsequent thrombocyte activation [118,119]. As a result of the activation and aggregation of platelets, an aggregate of thrombocytes is produced around the tumor cells [122,123,124,125]. Due to the TCIPA process, thrombocytes protect cancer cells and promote tumor promotion. The inhibition of TCIPA should be a new therapeutic target in cancer treatment, as it reduces the cancer’s ability to form secondary foci in the body [122,123]. Importantly, the synthesized OGF-Gem conjugate inhibited AsPC-1-induced platelet aggregation at a concentration that was non-toxic against blood platelets. Additionally, for the first time, it has been demonstrated that OGF also influences the inhibition of the TCIPA process. It has to be noticed that Gem, one of the most commonly used chemotherapeutics for pancreatic cancer treatment, did not affect TCIPA. In our research, the TCIPA process was stimulated solely by the presence of pancreatic cancer cells, without the addition of 1% platelet-poor plasma. The studies were conducted on washed platelets suspended in Tyrode buffer, which confirmed the aggressive nature of the AsPC-1 cancer cells [118,126,127]. The absence of the TCIPA process induction by MIA PaCa-2 cells may be associated, as suggested by Haschemi et al., with low levels of ligands for P-selectin and tissue factor, leading to the inability to form an aggregate with platelets [118]. 

The obtained results indicate the potential for utilizing the novel OGF-Gem conjugate in the development of safe drugs that may inhibit the metastasis of oncological diseases, in contrast to the commonly used chemotherapeutic agents such as gemcitabine, which also demonstrate high cytotoxicity against non-tumor transformed cells.

## 5. Conclusions

In summary, herein, we presented the design and synthesis of a novel peptide–drug conjugate, OGF-Gem, specifically tailored to target pancreatic ductal adenocarcinoma. OGF-Gem demonstrates several potent preclinical elements that deserve further evaluation in cancer research. Specifically, this novel peptide–drug conjugate (1) is cytotoxic against pancreatic lines, including one metastatic line, (2) reduces the viability of patient-derived tumor organoids, and (3) shows a significantly lower cytotoxicity for non-tumorous transformed cells, as opposed to Gem exposure. It also (4) induces proliferation and (5) cell cycle inhibition in MIA PaCa-2 and AsPC-1 cells, and (6) induces senescence in all pancreatic cancer lines tested. Finally, it (7) induces a particular type of cell death depending on the lineage apoptosis induced in MIA PaCa-2 cells, and (8) inhibits platelet aggregation induced by AsPC-1 cells. Further investigations of its mechanism of action and pharmacological activity should be conducted. The obtained results suggest that the OGF-Gem conjugate plays a role in influencing the promotion and progression of pancreatic cancer. However, to gain a deeper understanding of its mechanism of action and pharmacological activity, further investigations are warranted. Further studies, encompassing a mouse tumor xenograft model, are crucial steps to solidify and validate the anti-cancer properties of OGF-Gem before venturing into potential clinical applications.

## Figures and Tables

**Table 1:** Cytotoxic effect of OGF-Gem conjugate against pancreatic cancer cells, MIA PaCa-2 and AsPC-1 after 72 h of incubation. Data are mean ± SD of three separate determinations. IC_50_ and logIC_50_ values were calculated based on the MTT cytotoxicity test.

Cell Line	IC_50_ ± SD	logIC_50_ ± SD
MIA PaCa-2	17.63 ± 2.334	1.25 ± 0.37
AsPC-1	27.44 ± 9.161	1.44 ± 0.96

## Data Availability

Data are contained within the article.

## Supplementary Materials

The following supporting information can be downloaded at https://www.mdpi.com/article/10.3390/pharmaceutics16020283/s1, Figure S1. (A) RP-HPLC analysis of compound 2 (tR = 11.75 min). (B) 1H NMR spectrum of compound **2** in DMSO-d6 at 298 K; (C) 13C NMR spectrum of compound **2** in DMSO-d6 at 298 K. (D) RP-HPLC analysis of the OGF. (E) RP-HPLC analysis of the OGF-Gem conjugate. (F) MS (MALDI) spectrum of OGF-Gem conjugate; *m*/*z* for C40H48F2N8O13S: calcd, 918.3; found, found signals 919.338 ([M + H]+, signal intensity 761,746), 941.315 ([M + Na]+, signal intensity 340,191), and 957.389 ([M + K]+, signal intensity 518,404). CCA. The remaining visible signals, such as 712.214, 979.274 and 1029.334 have significantly lower intensities 41,852, 53,856 and 2395, respectively; Figure S2. HPLC chromatograms and MS spectra obtained for OGF-Gem conjugate before (A) and after incubation with medium (DMEM) supplemented with 10% FBS. The sample collected immediately after the start of incubation (B), 30 min (C), 90 min (D), 180 min (E), and 23 h (F). Chromatogram for DMEM is shown as (G). Linear gradient 10–90% phase B, 20 min, 1 mL/min, column Kinetex 5 μm XB-C18 100Å 150 × 4.6 mm, 214 nm; Figure S3. HPLC chromatograms obtained in method: 0–5 min 0% phase B, 5–25 min gradient 0–60% of phase B, 1 mL/min, column Kinetex 5 μm XB-C18 100Å 150 × 4.6 mm, 214 nm. OGF-Gem conjugate alone (A); Gem (B); supplemented medium (C); OGF-Gem conjugate incubated for 3 days at room temperature with medium (DMEM) supplemented with 10% FBS (D); superimposed HPLC chromatograms (E) obtained in slightly modified method: 0–5 min 0% phase B, 5–20 min gradient 0–60%, 20–25 min 60% of phase B, 1 mL/min for OGF-Gem conjugate alone (brown), Gem (green), supplemented medium (red) and OGF-Gem incubated with medium (blue); Figure S4. HPLC chromatograms and MS spectra obtained for OGF before (A) and after incubation with medium (DMEM), supplemented with 10% FBS immediately after the start of incubation (B), 30 min (C), 90 min (D), 180 min (E), and 23 h (F). Linear gradient 10–90% phase B, 20 min, 1 mL/min, column Kinetex 5 μm XB-C18 100Å 150 × 4.6 mm, 214 nm; Figure S5. Effect of pancreatic cancer cells MIA PaCa-2 on platelets aggregation (TCIPA). (A) Representative LTA graph showing TCIPA induced by MIA PaCa-2 cells depending on the concentration. (B) Data are mean ± SD of 5 separate determinations.
