# Supplementary material for: Design, Synthesis, and Antitumor Evaluation of an Opioid Growth Factor Bioconjugate Targeting Pancreatic Ductal Adenocarcinoma
Source: Pharmaceutics. 2024 Feb 16;16(2):283. doi: 10.3390/pharmaceutics16020283 (PMC10892429; doi:10.3390/pharmaceutics16020283)
Supplement: Supplementary file 1 [file pharmaceutics-16-00283-s001.zip › pharmaceutics-2835495-supplementary.pdf]

Supplementary File

Synthesis, characterization, and evaluation of potential anti-tumor activity of Opioid Growth Factor derivative against pancreatic ductal adenocarcinoma cells

Justyna Budka [1], Dawid Debowski [2], Shaoashan Mai [1], Magdalena Narajczyk [3], Stanislaw Hac [4], Krzysztof Rolka [2], Eirinaios I. Vrettos [5], Andreas G. Tzakos [5,6] and Iwona Inkielewicz-Stepniak [1*]

1. **Supplementary Figures**

(A)

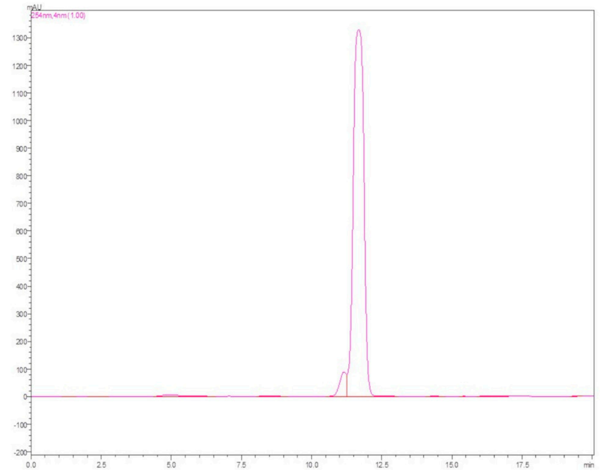

(B)

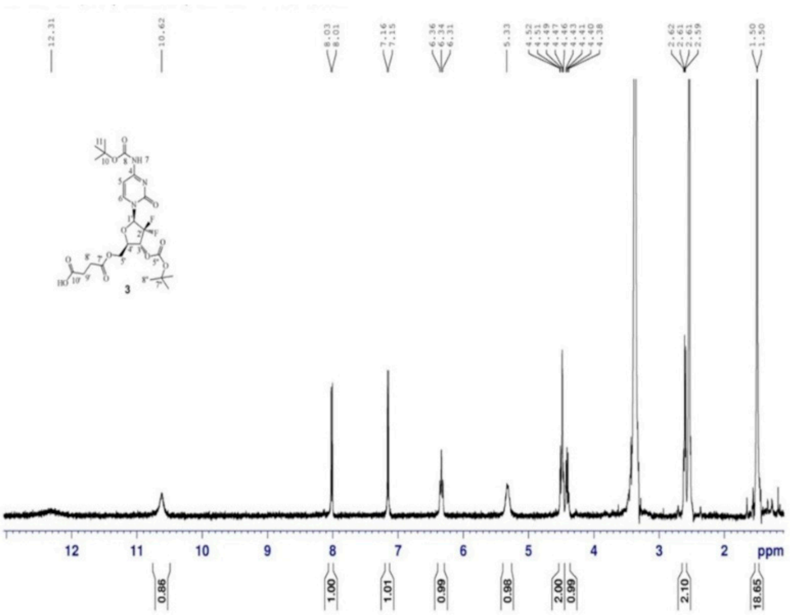

(C)

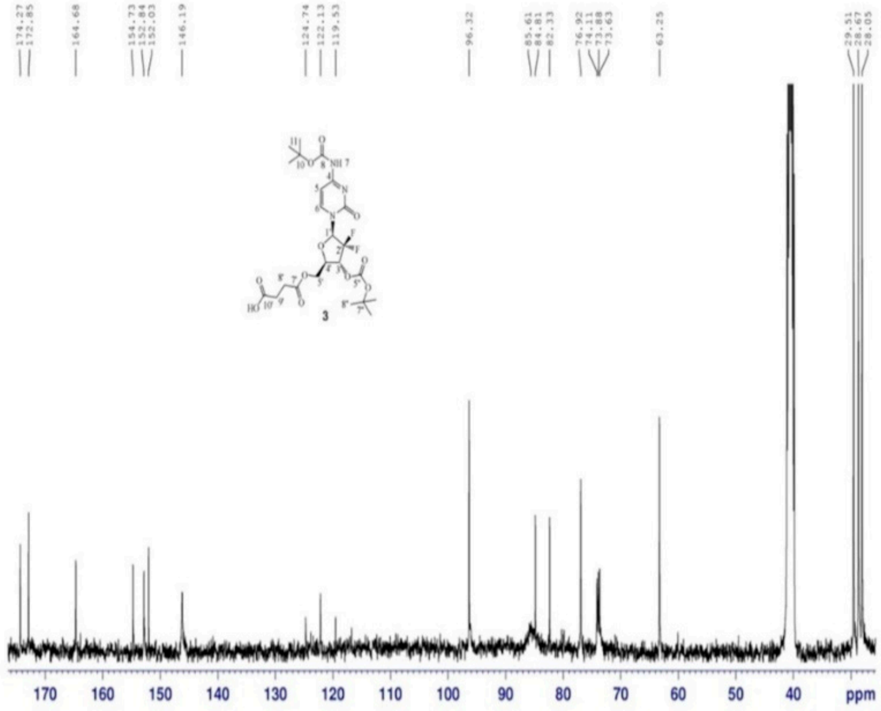

174.27
172.85
164.68
154.73
152.84
152.03
146.19
124.74
122.13
119.53
96.32
85.61
84.81
82.33
76.92
74.11
73.88
73.63
63.25
29.51
28.67
28.05
3
170 160 150 140 130 120 110 100 90 80 70 60 50 40
ppm

(D)

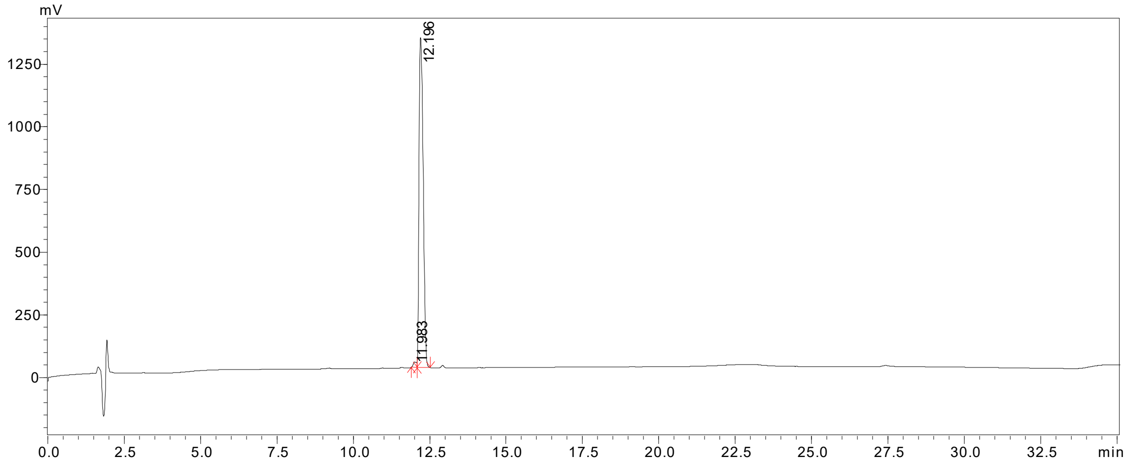

mV
1250
1000
750
500
250
0
12.196
11.983
0.0 2.5 5.0 7.5 10.0 12.5 15.0 17.5 20.0 22.5 25.0 27.5 30.0 32.5
min

(E)

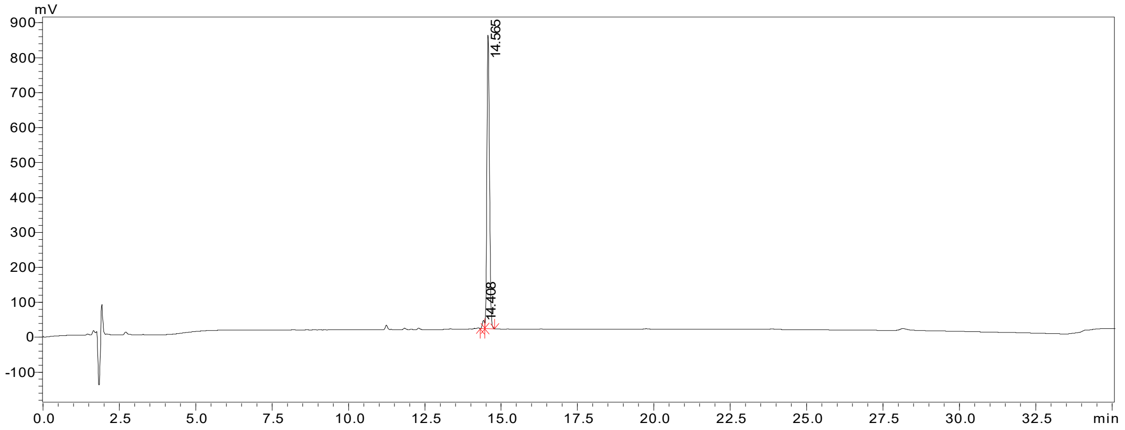

(F)

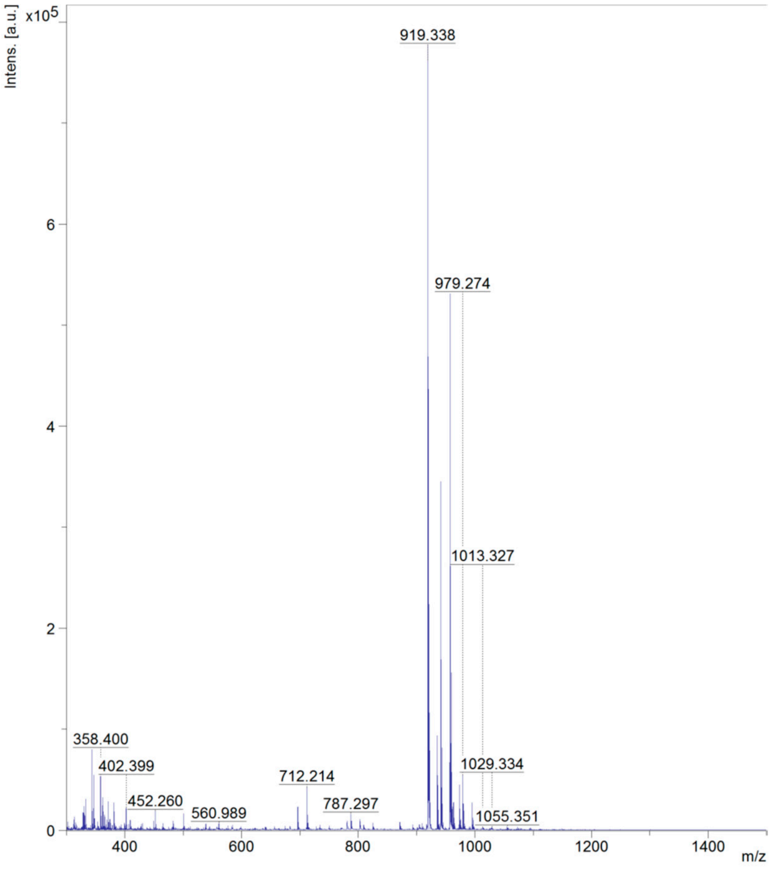

Supplementary Figure S1. (A) RP-HPLC analysis of compound 2 (tR = 11.75 min). (B) 1H NMR spectrum of compound 2 in DMSO-d6 at 298 K; (C) 13C NMR spectrum of compound 2 in DMSO-d6 at 298 K. (D) RP-HPLC analysis of the OGF. (E) RP-HPLC analysis of the OGF-Gem conjugate. (F) MS (MALDI) spectrum of OGF-Gem conjugate; m/z for C40H48F2N8O13S: calcd, 918.3; found, found signals 919.338 ([M + H]+, signal intensity 761746), 941.315 ([M + Na]+, signal intensity 340191), and 957.389 ([M + K]+, signal intensity 518404). CCA. The

remaining visible signals, such as 712.214, 979.274 and 1029.334 have significantly lower intensities 41852, 53856 and 2395, respectively.

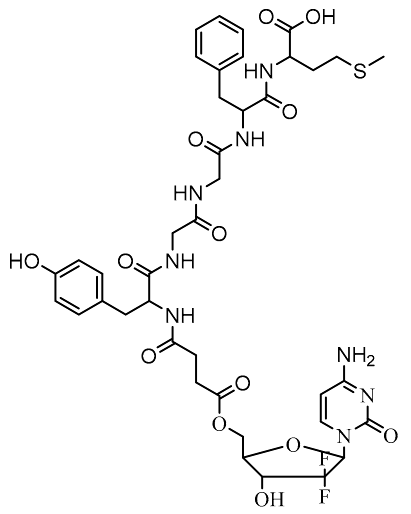

OGF-Gem, calculated exact mass 918.30

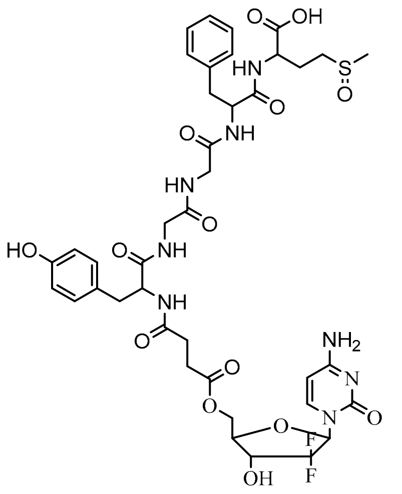

OGF-Gem with sulfoxide residues Met(O), calculated exact mass 934.30

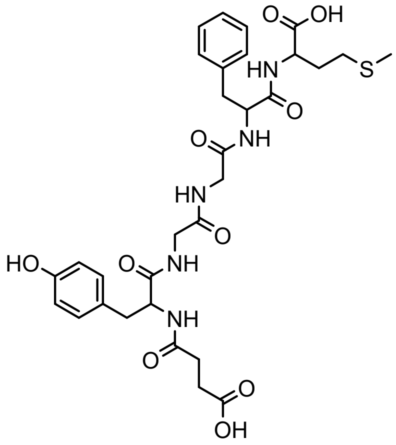

OGF with succinate linker, calculated exact mass 673.24 (found after 90 min of incubation)

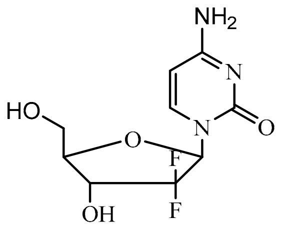

Gem calculated exact mass 263.07 (found after 90 min of incubation)

(A)

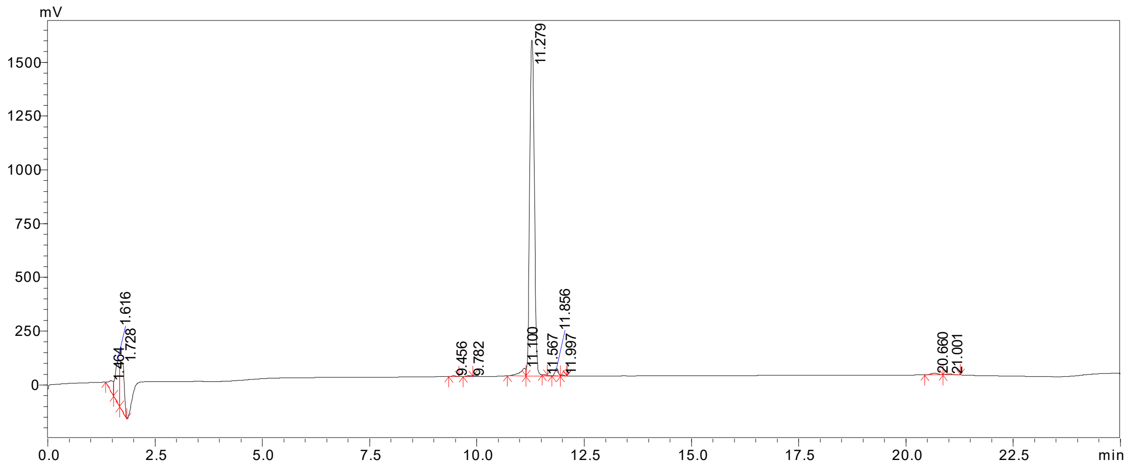

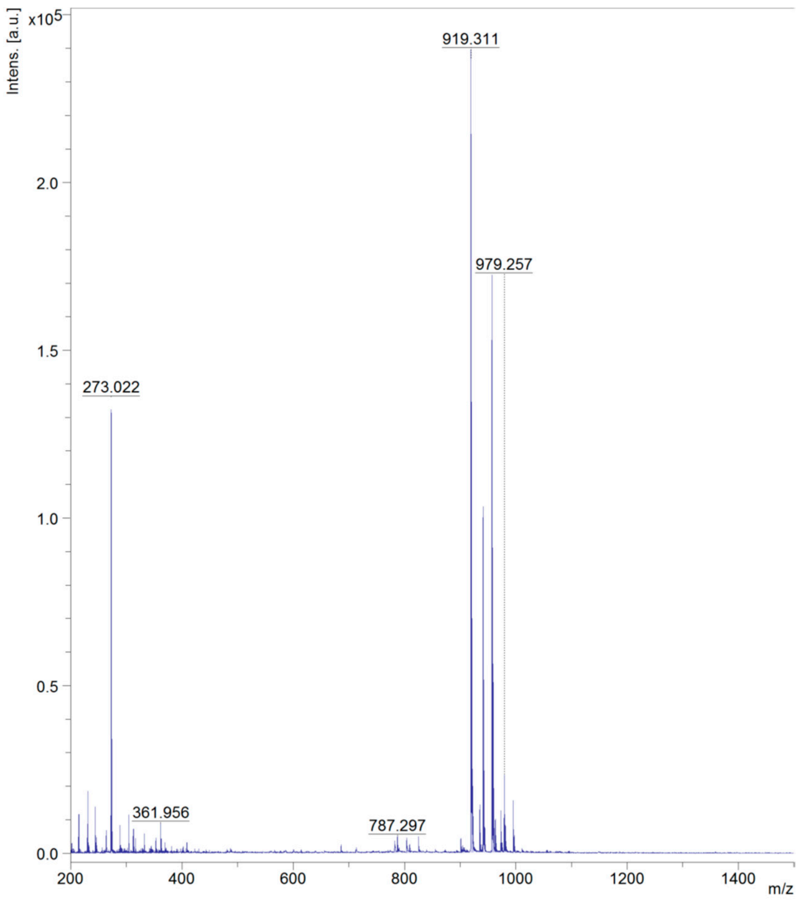

OGF-Gem calculated signals 919.30 [M+H]$^+$; 941.28 [M+Na]$^+$; 957.26 [M+K]$^+$
OGF-Gem found signals 919.31 (intensity 236827, area 133718); 941.28 (intensity 102056, area 40728); 957.26 (intensity 169165, area 81042);

OGF-Gem with Met(O) calculated signals 935.30 [M+H]$^+$; 957.28 [M+Na]$^+$; 973.26 [M+K]$^+$
OGF-Gem with Met(O) found signals 935.30 (intensity 14167, area 4714); 957.26 (intensity 169165, area 81042); 973.25 (intensity 12504, area 5501)

(B)

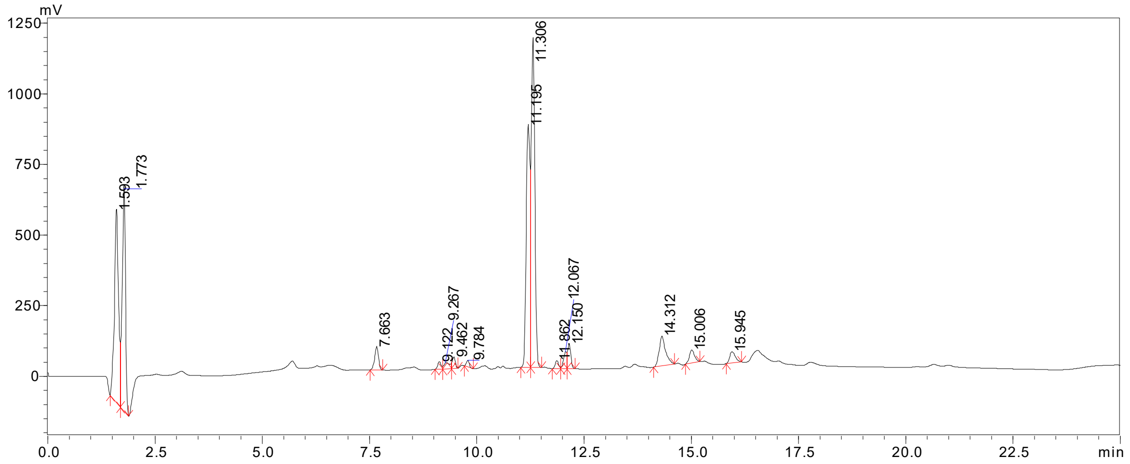

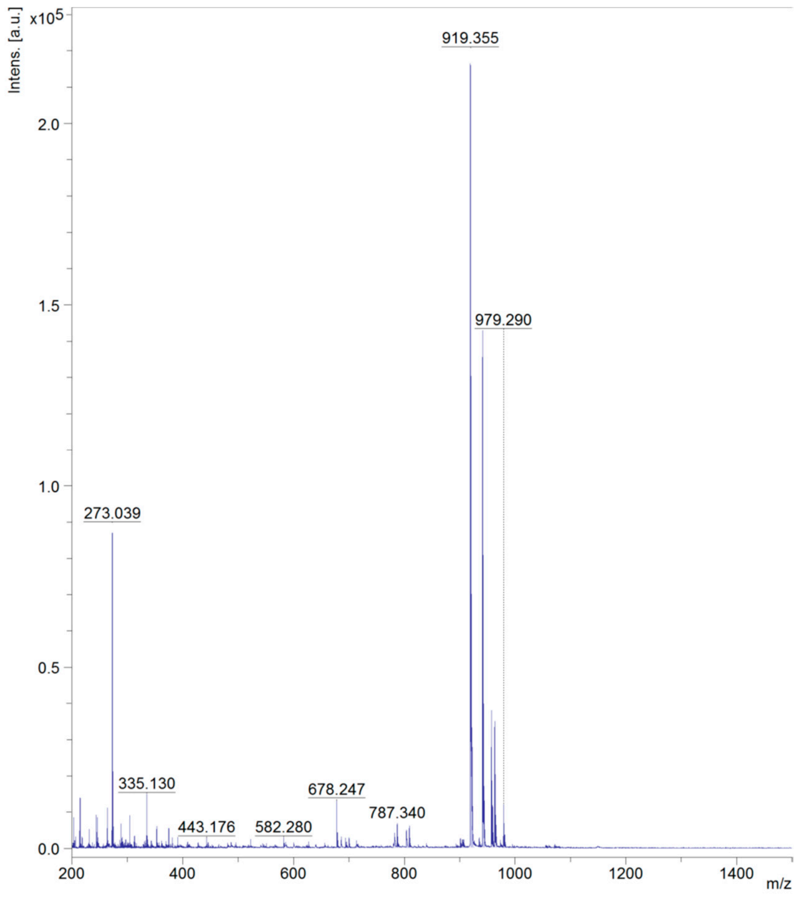

OGF-Gem calculated signals 919.30 $[M+H]^+$; 941.28 $[M+Na]^+$; 957.26 $[M+K]^+$
OGF-Gem found signals 919.35 (intensity 2206171, area 179895); 941.34 (intensity 147987, area 81400); 957.32 (intensity 38367, area 16442);

OGF-Gem with Met(O) calculated signals 935.30 $[M+H]^+$; 957.28 $[M+Na]^+$; 973.26 $[M+K]^+$
OGF-Gem with Met(O) found signals 935.37 (intensity 2861, area 1549); 957.32 (intensity 38367, area 16442); 973.36 (intensity 19121, area 939)

(C)

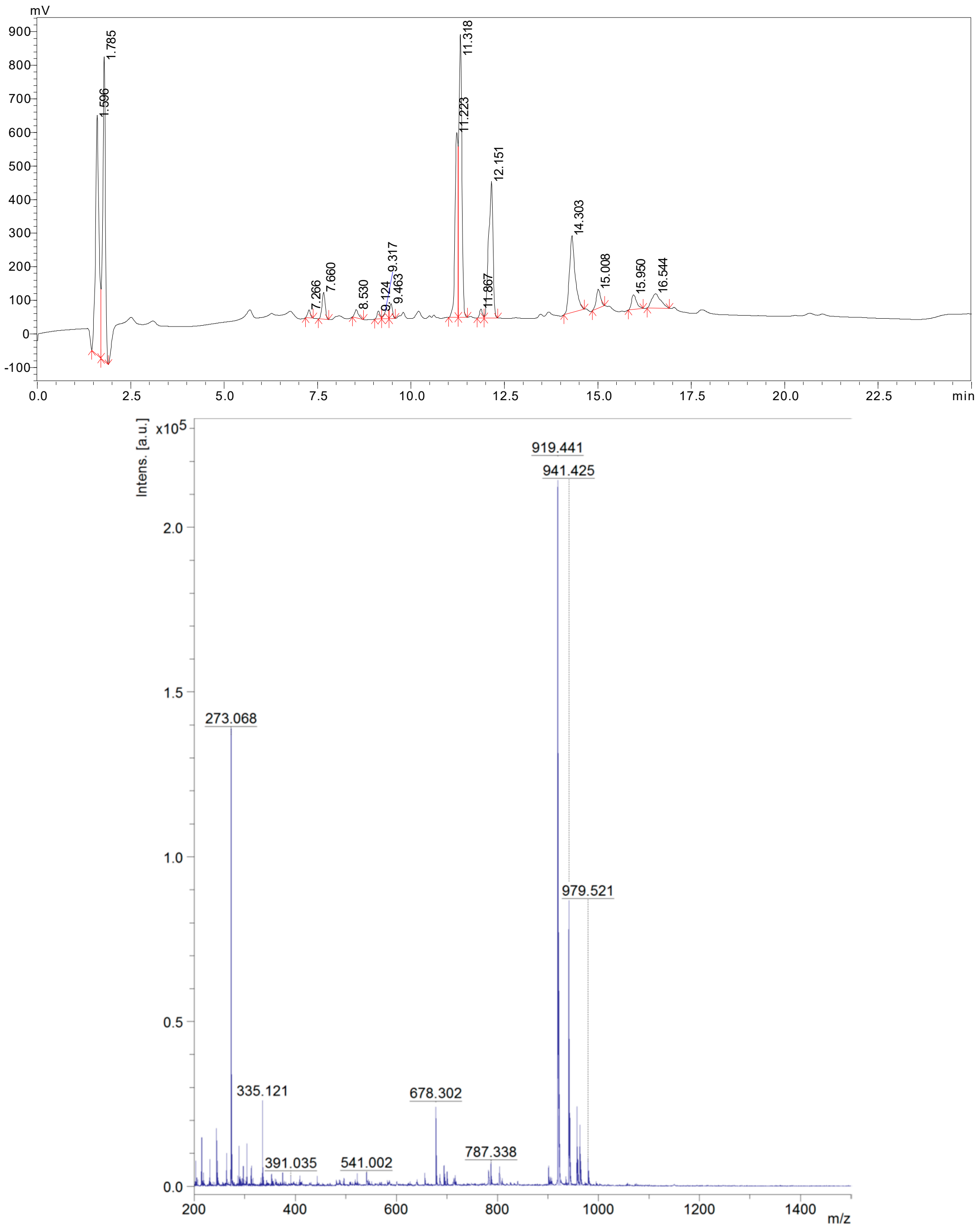

OGF-Gem calculated signals 919.30 [M+H]$^+$; 941.28 [M+Na]$^+$; 957.26 [M+K]$^+$
OGF-Gem found signals 919.44 (intensity 221238, area 211207); 941.42 (intensity 91632, area 70481); 957.49 (intensity 23087, area 17718);

OGF-Gem with Met(O) calculated signals 935.30 [M+H]$^+$; 957.28 [M+Na]$^+$; 973.26 [M+K]$^+$
OGF-Gem with Met(O) found signals 935.39 (intensity 2607, area 1619); 957.49 (intensity 23087, area 17718)

(D)

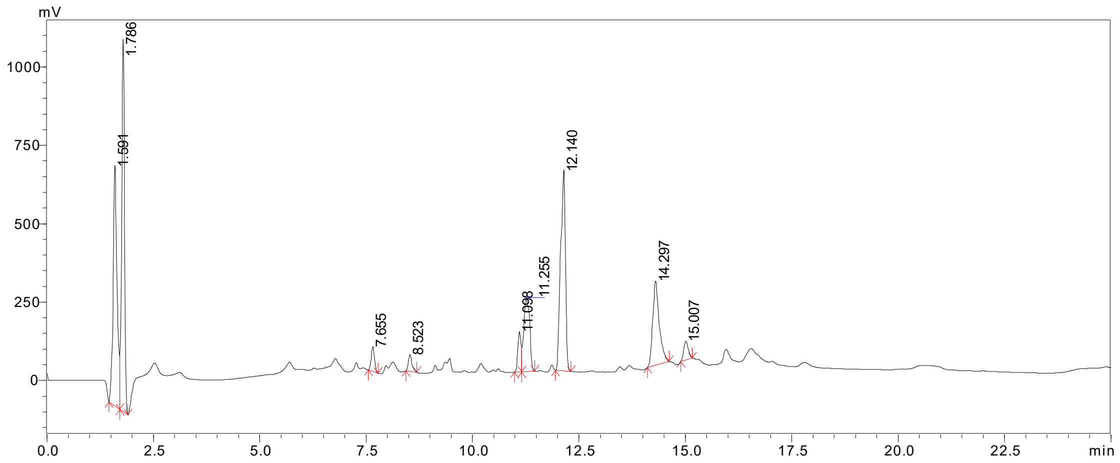

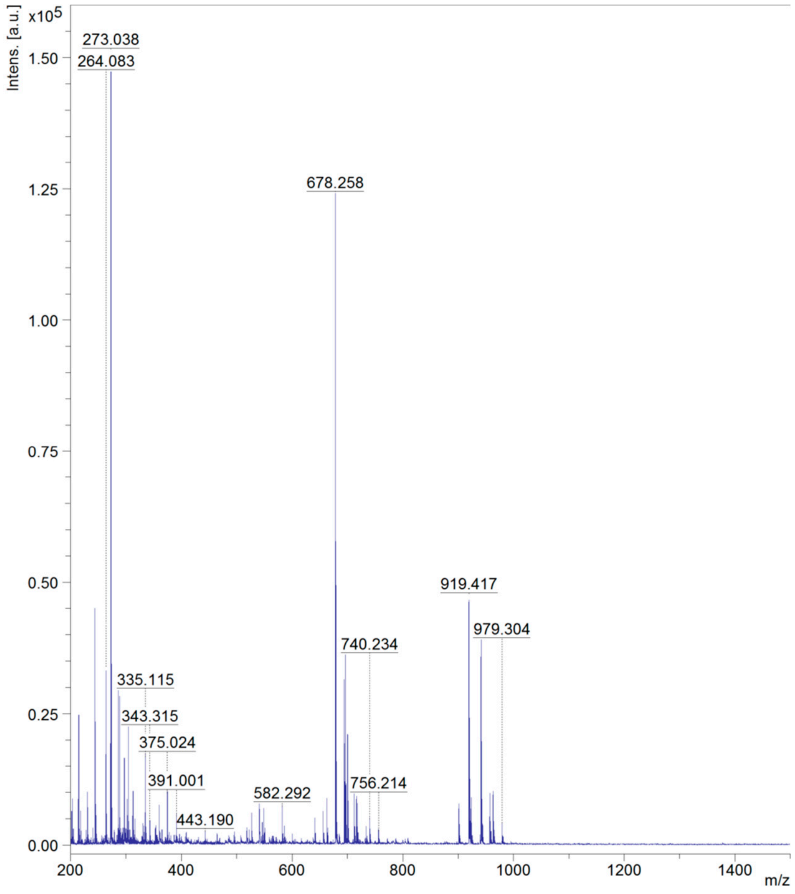

OGF-Gem calculated signals 919.30 $[M+H]^+$; 941.28 $[M+Na]^+$; 957.26 $[M+K]^+$
OGF-Gem found signals 919.41 (intensity 47655, area 23349); 941.35 (intensity 38926, area 17714); 957.32 (intensity 9522, area 3778);

OGF-Gem with Met(O) calculated signals 935.30 $[M+H]^+$; 957.28 $[M+Na]^+$; 973.26 $[M+K]^+$
OGF-Gem with Met(O) found signal 957.32 (intensity 9522, area 3778);

OGF with succinate linker calculated signals 674.20 [M+H]$^+$; 696.18 [M+Na]$^+$; 712.16 [M+K]$^+$
OGF with succinate linker found signals 696.126 (intensity 32333, area 9490); 712.24 (intensity 9477, area 2391);
Gem calculated signals 264.07 [M+H]$^+$; 286.05 [M+Na]$^+$; 302.03 [M+K]$^+$
Gem found signals 264.08 (intensity 33793, area 4890); 286.06 (intensity 28580, area 3565); 302.04 (intensity 6990, area 1414);

(E)

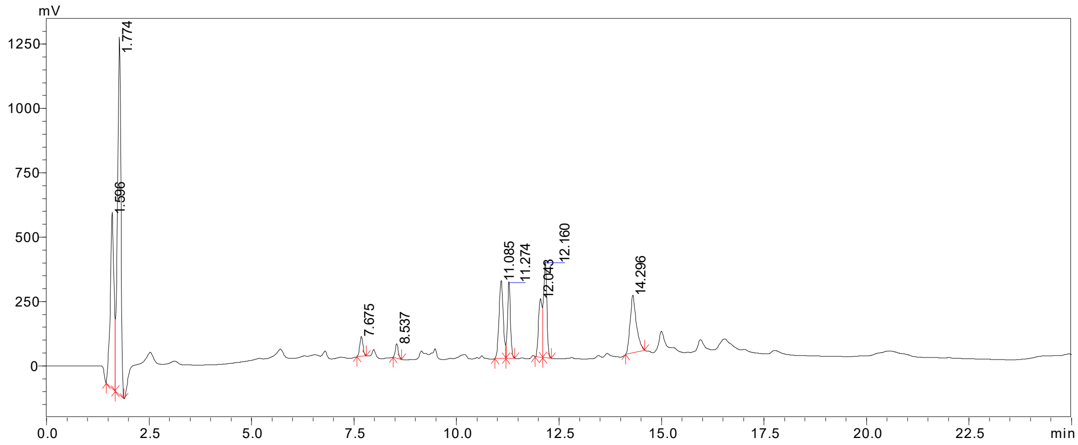

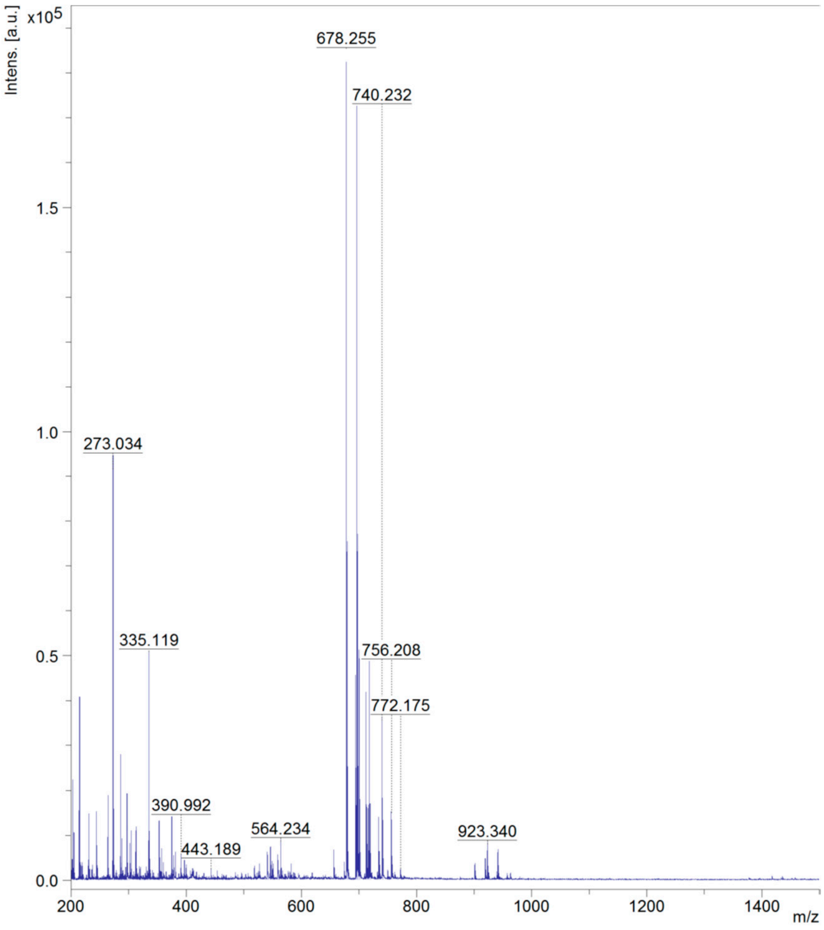

OGF-Gem calculated signals 919.30 [M+H]$^+$; 941.28 [M+Na]$^+$; 957.26 [M+K]$^+$

OGF-Gem found signals 919.38 (intensity 4660, area 1947); 941.34 (intensity 6377, area 2388)

OGF-Gem with Met(O) calculated signals 935.30 $[M+H]^+$; 957.28 $[M+Na]^+$; 973.26 $[M+K]^+$
OGF-Gem with Met(O) lack of signals

OGF with succinate linker calculated signals 674.20 $[M+H]^+$; 696.18 $[M+Na]^+$; 712.16 $[M+K]^+$
OGF with succinate linker found signals 674.29 (intensity 3894, area 1131); 696.26 (intensity 174876, area 52697); 712.25 (intensity 41412, area 11619)

Gem calculated signals 264.07 $[M+H]^+$; 286.05 $[M+Na]^+$; 302.03 $[M+K]^+$
Gem found signals 264.08 (intensity 18717, area2619); 286.06 (intensity 26889, area 3346); 302.03 (intensity 7396, area 1250);

(F)

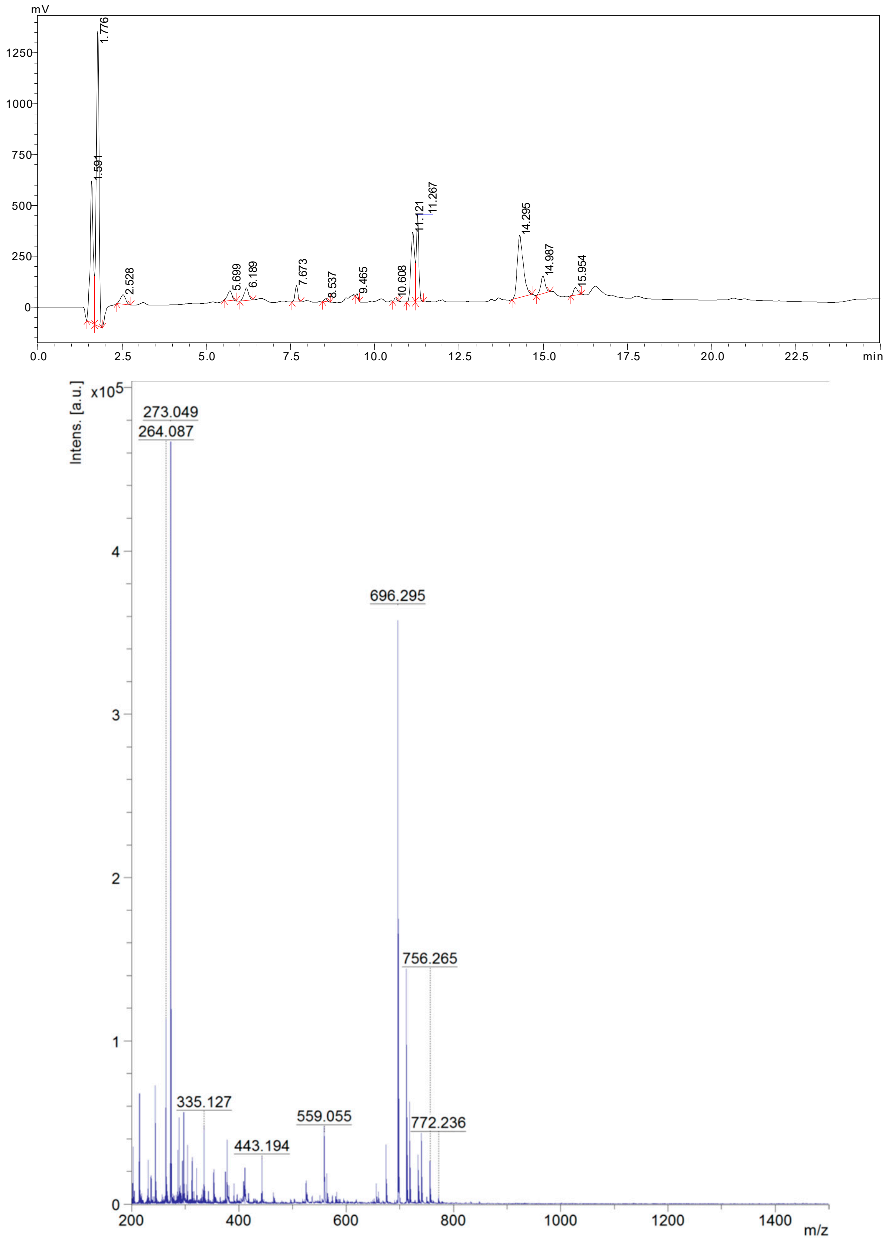

OGF-Gem calculated signals 919.30 [M+H]+ ; 941.28 [M+Na]+ ; 957.26 [M+K]+
OGF-Gem lack of signals

OGF-Gem with Met(O) calculated signals 935.30 [M+H]+ ; 957.28 [M+Na]+ ; 973.26 [M+K]+
OGF-Gem with Met(O) lack of signals

OGF with succinate linker calculated signals 674.20 [M+H]+ ; 696.18 [M+Na]+ ; 712.16 [M+K]+
OGF with succinate linker found signals 674.32 (intensity 37026, area 13835); 696.29 (intensity 366432, area 145843); 712.28 (intensity 146377, area 52769)

Gem calculated signals 264.07 [M+H]+ ; 286.05 [M+Na]+ ; 302.03 [M+K]+
Gem found signals 264.08 (intensity 113401, area 18081); 286.06 (intensity 30956, area 5713); 302.04 (intensity 10319, area 3510);

(G)

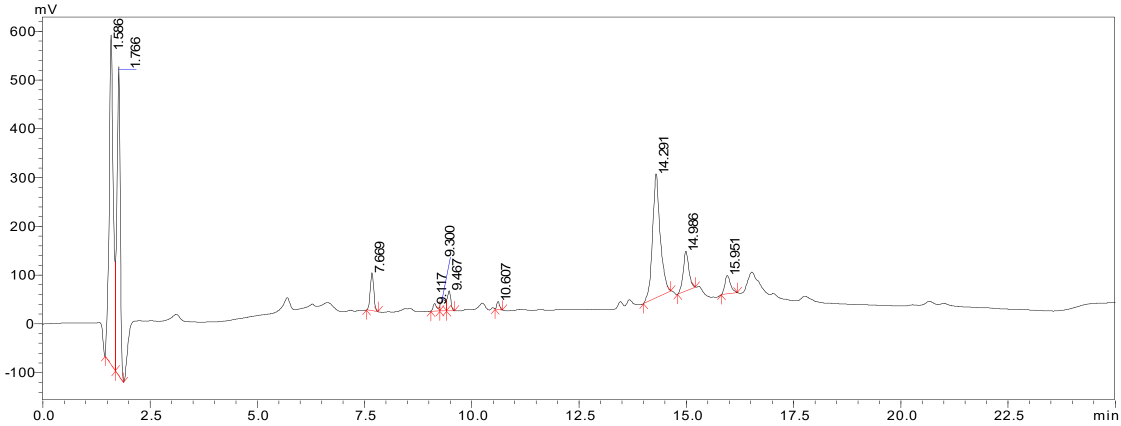

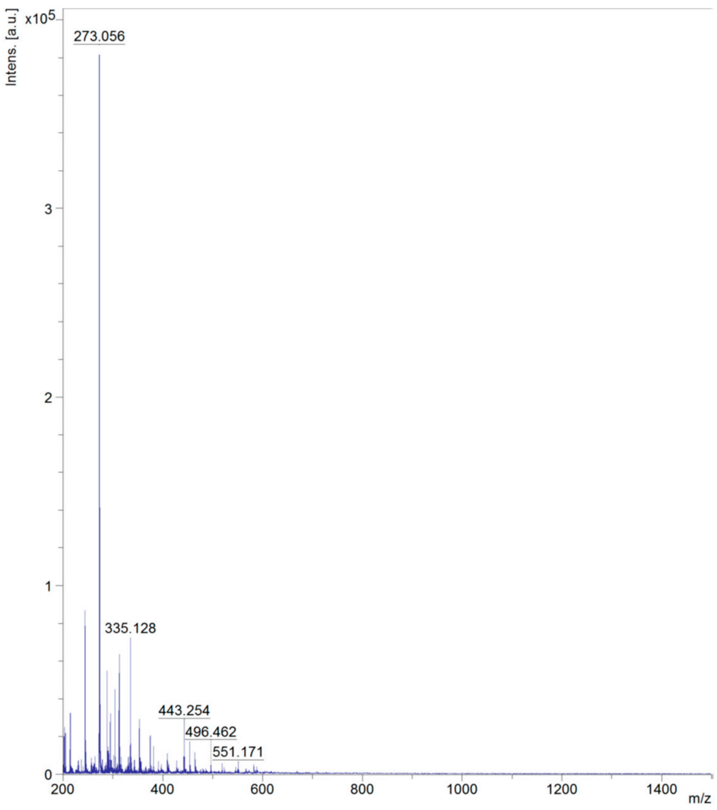

Supplementary Figure S2. HPLC chromatograms and MS spectra obtained for OGF-Gem conjugate before (A) and after incubation with medium (DMEM) supplemented with 10% FBS. The sample collected immediately after the start of incubation (B), 30 min (C), 90 min (D), 180 min (E), and 23 hours (F). Chromatogram for DMEM is shown as (G). Linear gradient 10-90% phase B, 20 min., 1 ml/min, column Kinetex 5 µm XB-C18 100Å 150 x 4.6 mm, 214 nm.

(A)

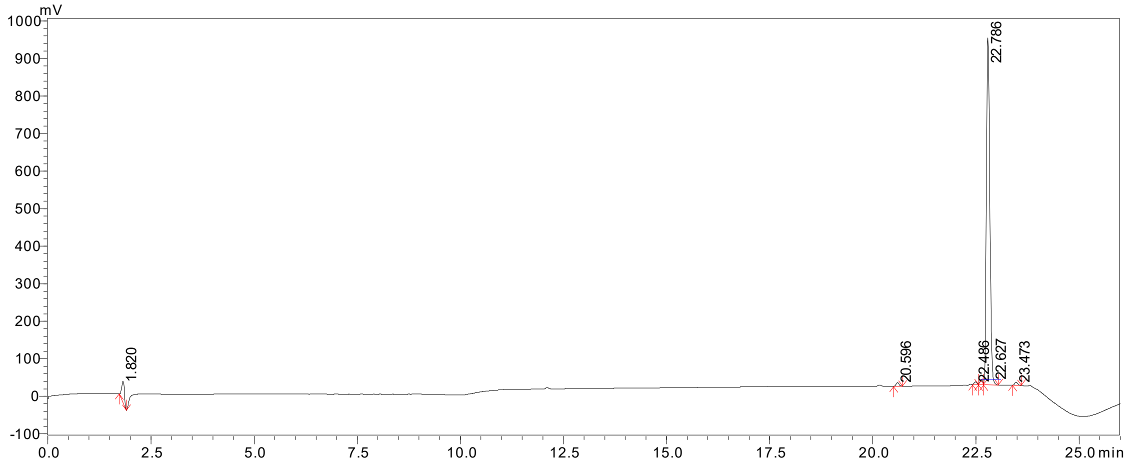

(B)

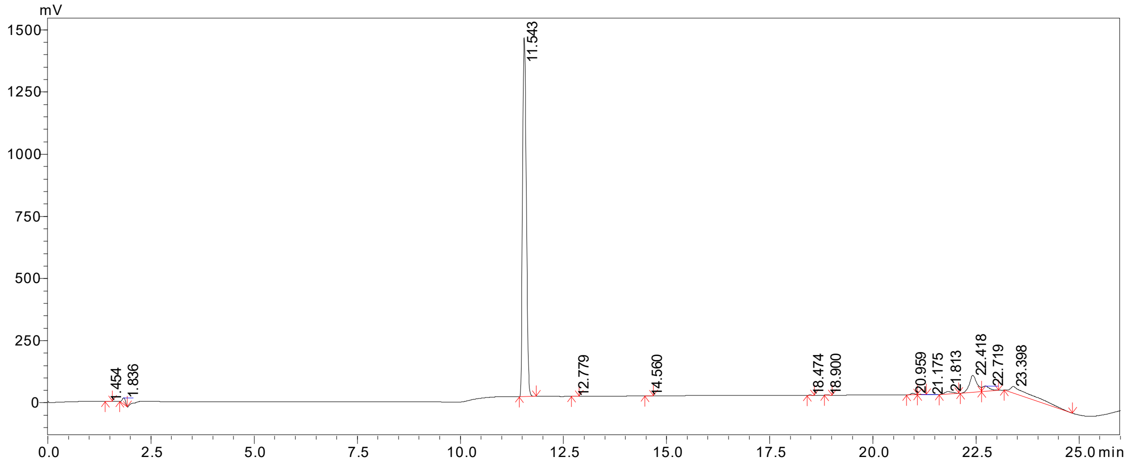
mV
1500
1250
1000
750
500
250
0
11.543
1.454
1.836
12.779
14.560
18.474
18.900
20.959
21.175
21.813
22.418
22.719
23.398
0.0
2.5
5.0
7.5
10.0
12.5
15.0
17.5
20.0
22.5
25.0 min

(C)

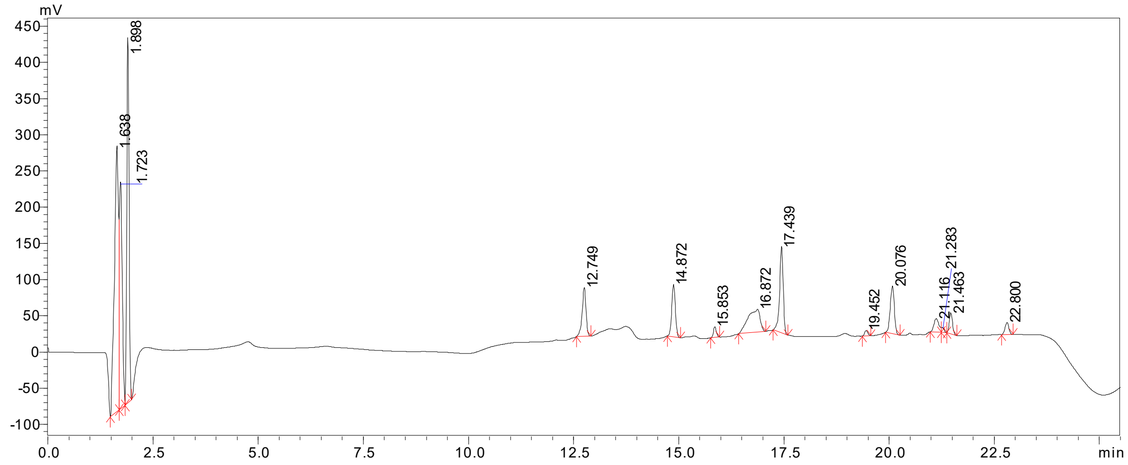
mV
450
400
350
300
250
200
150
100
50
0
-50
-100
1.898
1.638
1.723
12.749
14.872
15.853
16.872
17.439
19.452
20.076
21.116
21.283
21.463
22.800
0.0
2.5
5.0
7.5
10.0
12.5
15.0
17.5
20.0
22.5
min

(D)

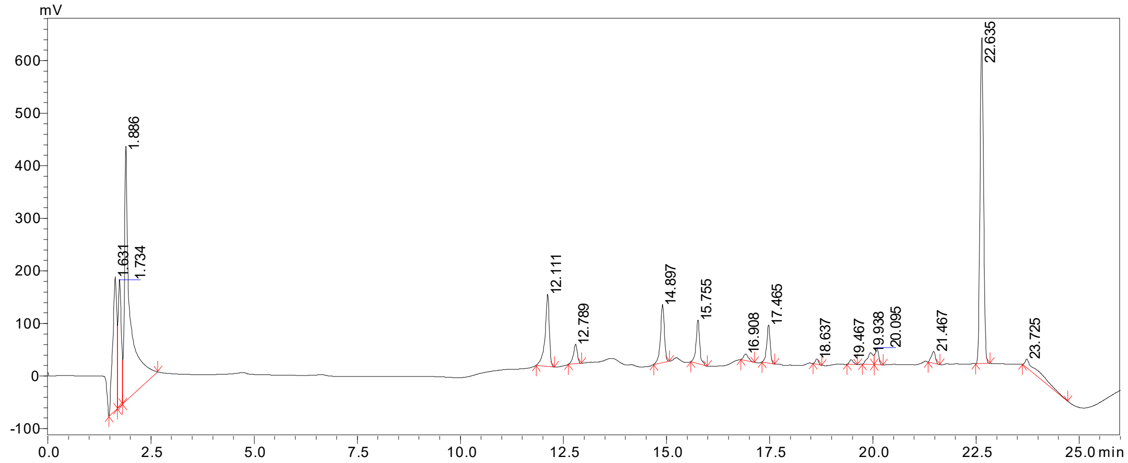
mV
600
500
400
300
200
100
0
-100
1.886
1.631
1.734
12.111
12.789
14.897
15.755
16.908
17.465
18.637
19.467
19.938
20.095
21.467
22.635
23.725
0.0
2.5
5.0
7.5
10.0
12.5
15.0
17.5
20.0
22.5
25.0 min

(E)

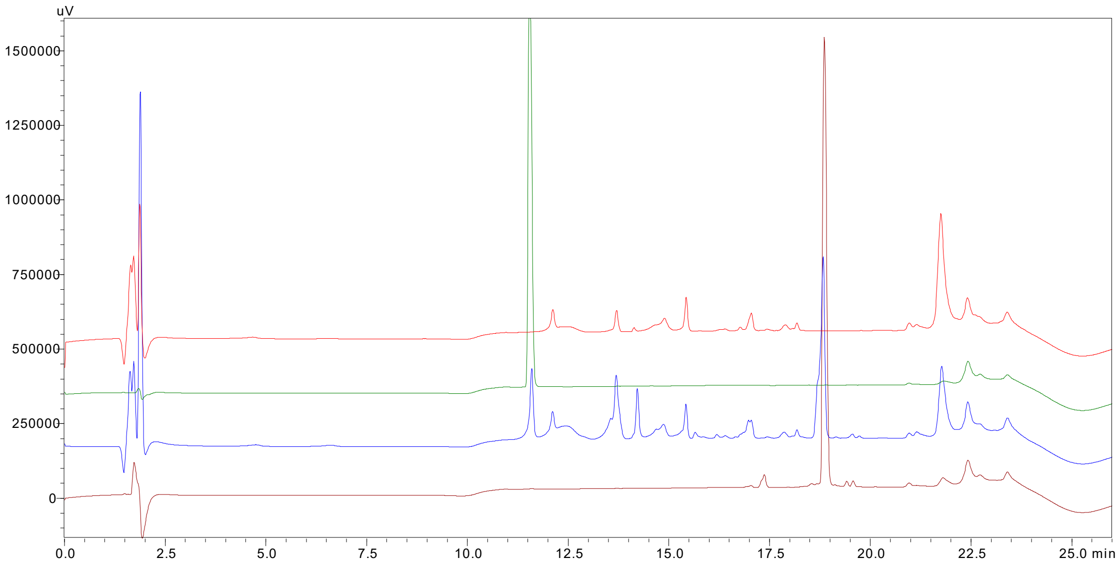

Supplementary Figure S3. HPLC chromatograms obtained in method: 0 - 5 min 0% phase B, 5 - 25 min gradient 0-60% of phase B, 1 ml/min, column Kinetex 5 µm XB-C18 100Å 150 x 4.6 mm, 214 nm. OGF-Gem conjugate alone (**A**); Gem (**B**); supplemented medium (**C**); OGF-Gem conjugate incubated for 3 days at room temperature with medium (DMEM) supplemented with 10% FBS (**D**); superimposed HPLC chromatograms (**E**) obtained in slightly modified method: 0 - 5 min 0% phase B, 5 - 20 min gradient 0-60%, 20-25 min 60% of phase B, 1 ml/min for OGF-Gem conjugate alone (**brown**), Gem (**green**), supplemented medium (**red**) and OGF-Gem incubated with medium (**blue**).

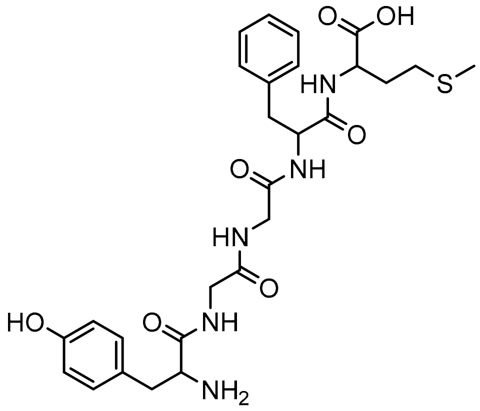

OGF, calculated exact mass 573.23

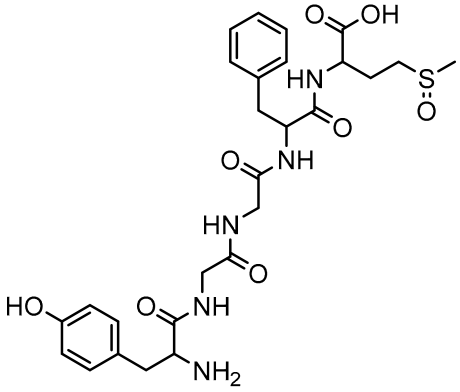

OGF with sulfoxide residues Met(O), calculated exact mass 589.22

(A)

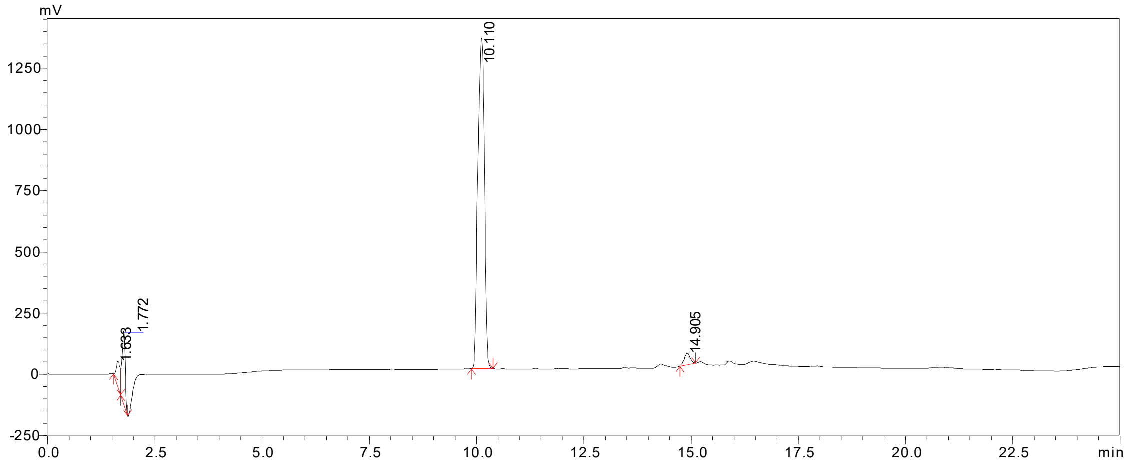

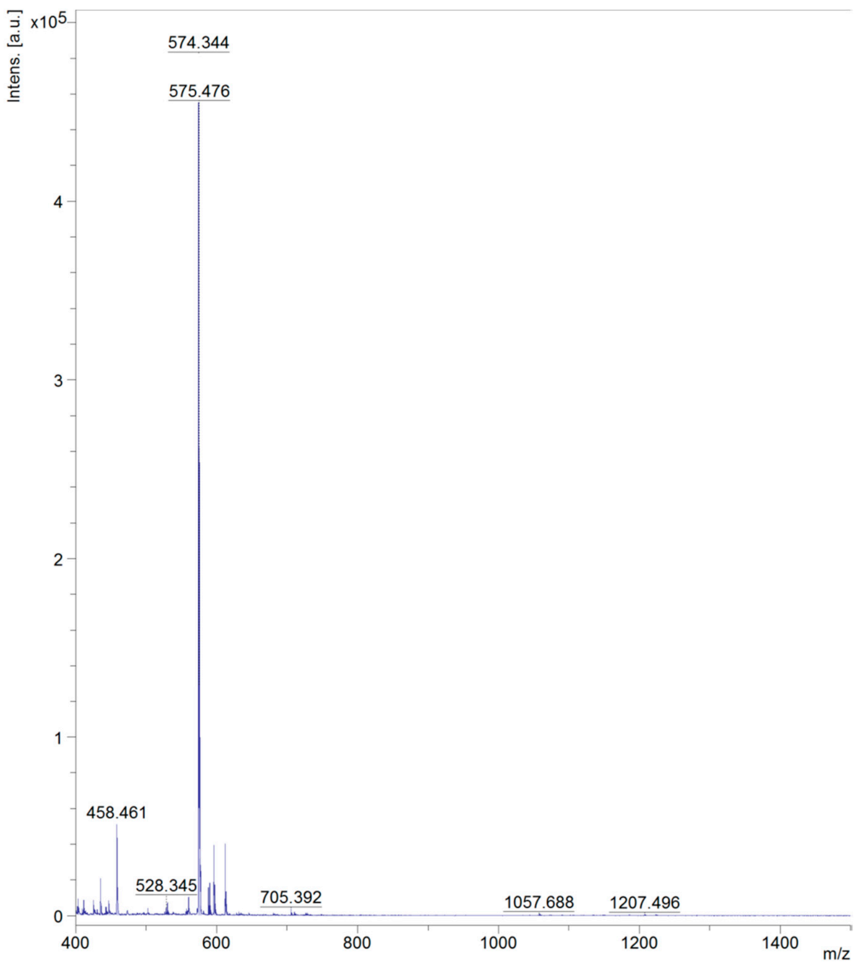

OGF calculated signals 574.23 $[M+H]^+$; 596.21 $[M+Na]^+$; 612.19 $[M+K]^+$

OGF found signals 574.34 (intensity 482301, area 388390); 596.26 (intensity 38865, area 20050); 612.25 (intensity 37079, area 20696);

OGF with Met(O) calculated signals 590.22 $[M+H]^+$; 612.20 $[M+Na]^+$; 628.18 $[M+K]^+$
OGF with Met(O) found signals 590.32 (intensity 17283, area 8331); 612.25 (intensity 37079, area 20696);

(B)

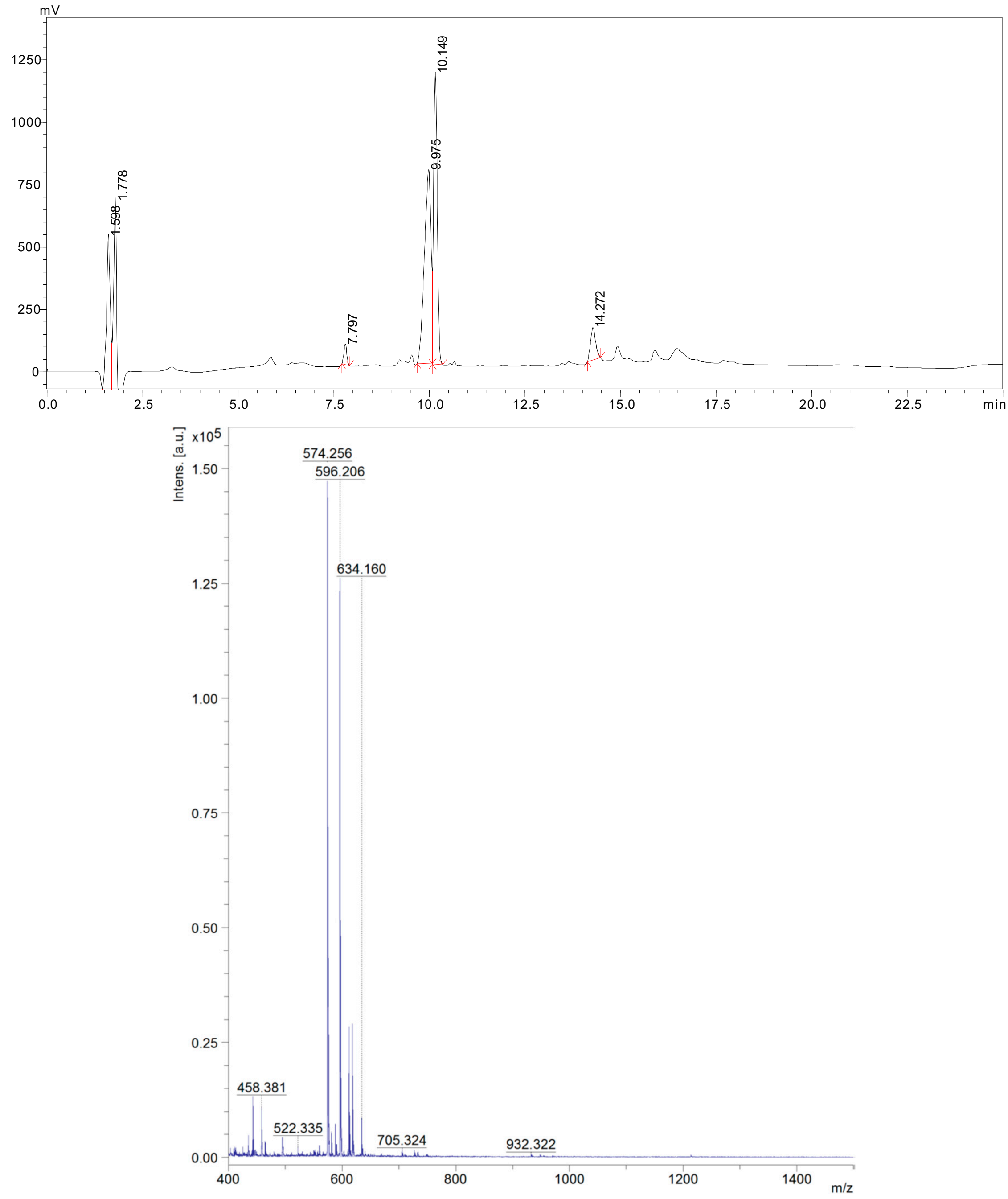

OGF calculated signals 574.23 $[M+H]^+$; 596.21 $[M+Na]^+$; 612.19 $[M+K]^+$

OGF found signals 574.25 (intensity 151252, area 98414); 596.20 (intensity 129707, area 69807); 612.19 (intensity 28020, area 13401);

OGF with Met(O) calculated signals 590.22 [M+H]+ ; 612.20 [M+Na]+ ; 628.18 [M+K]+
OGF with Met(O) found signals 590.25 (intensity 1993, area 1072); 612.19 (intensity 28020, area 13401);

(C)

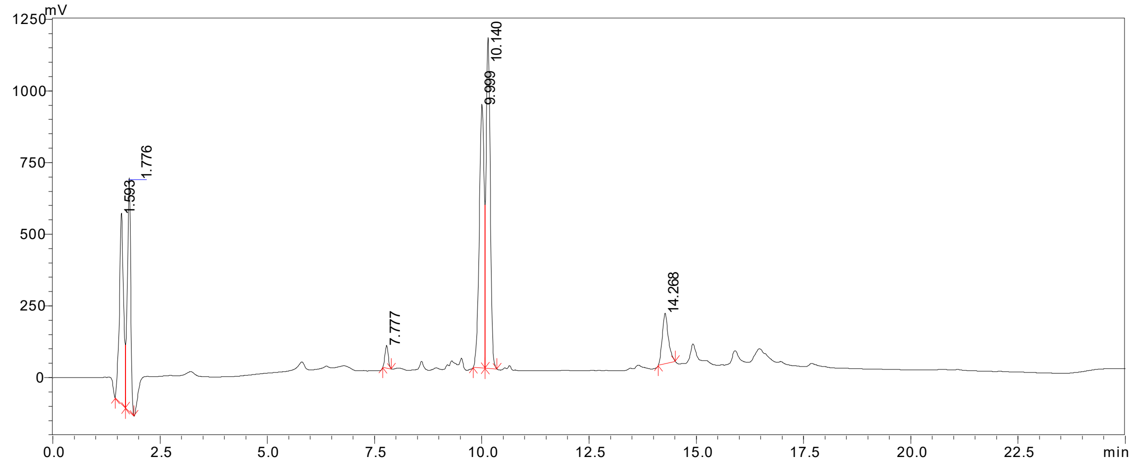

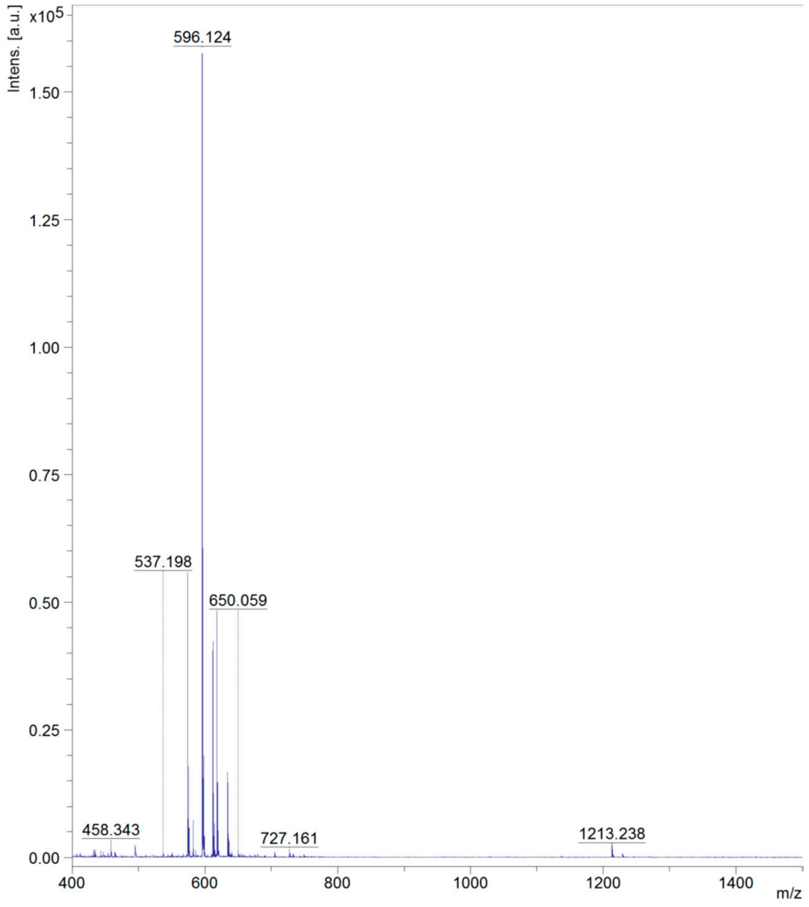

OGF calculated signals 574.23 [M+H]+ ; 596.21 [M+Na]+ ; 612.19 [M+K]+

OGF found signals 574.15 (intensity 55540, area 10820); 596.12 (intensity 158628, area 38952); 612.10 (intensity 42784, area 8288);
Lack of recorded signals corresponding to the OGF with Met(O)

(D)

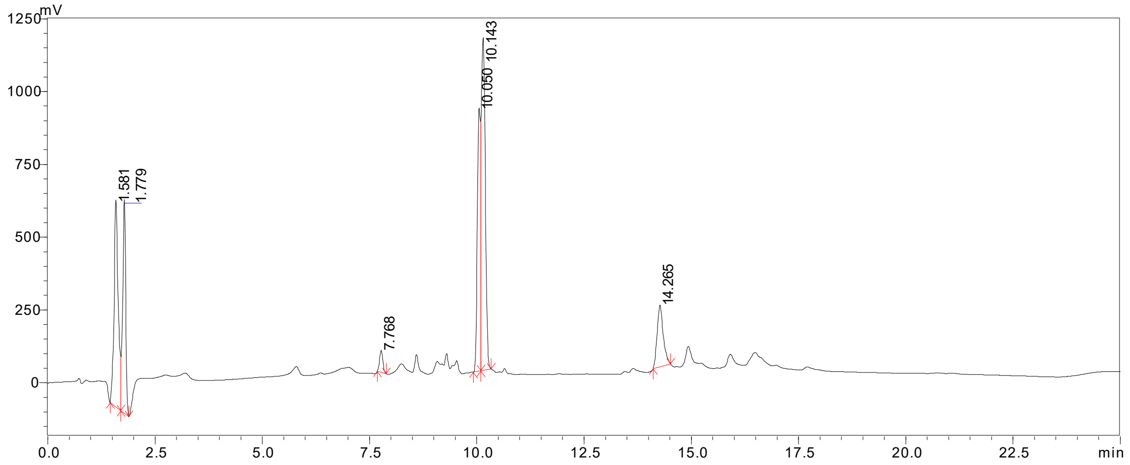

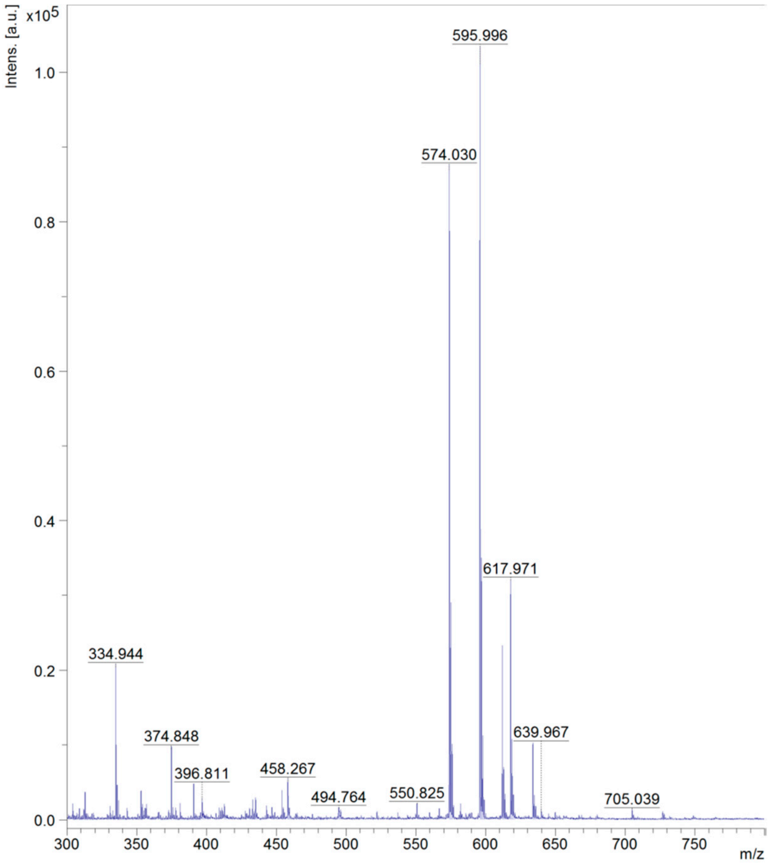

OGF calculated signals 574.23 $[M+H]^+$; 596.21 $[M+Na]^+$; 612.19 $[M+K]^+$
OGF found signals 574.03 (intensity 86861, area 27741); 595.99 (intensity 100983, area 34462); 611.97 (intensity 22262, area 6617);
Lack of recorded signals corresponding to the OGF with Met(O)

(E)

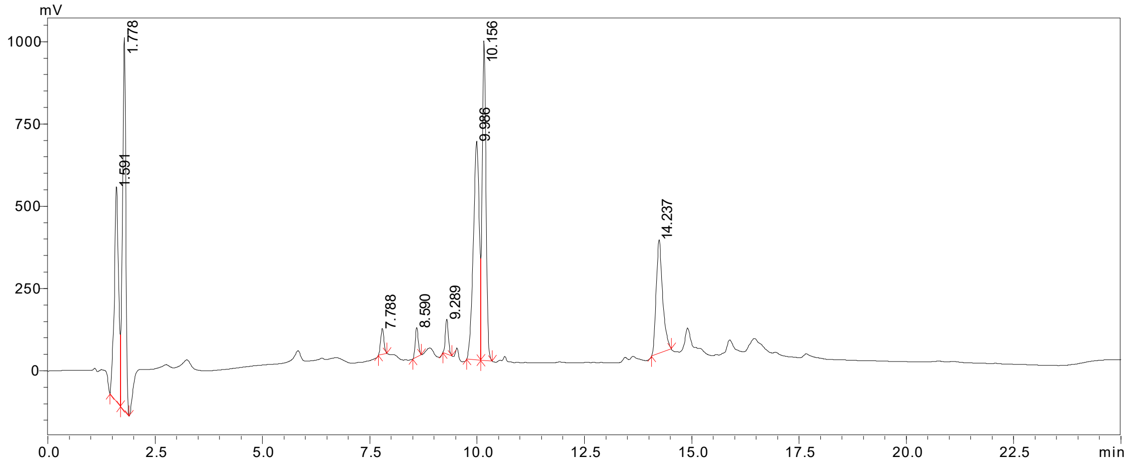

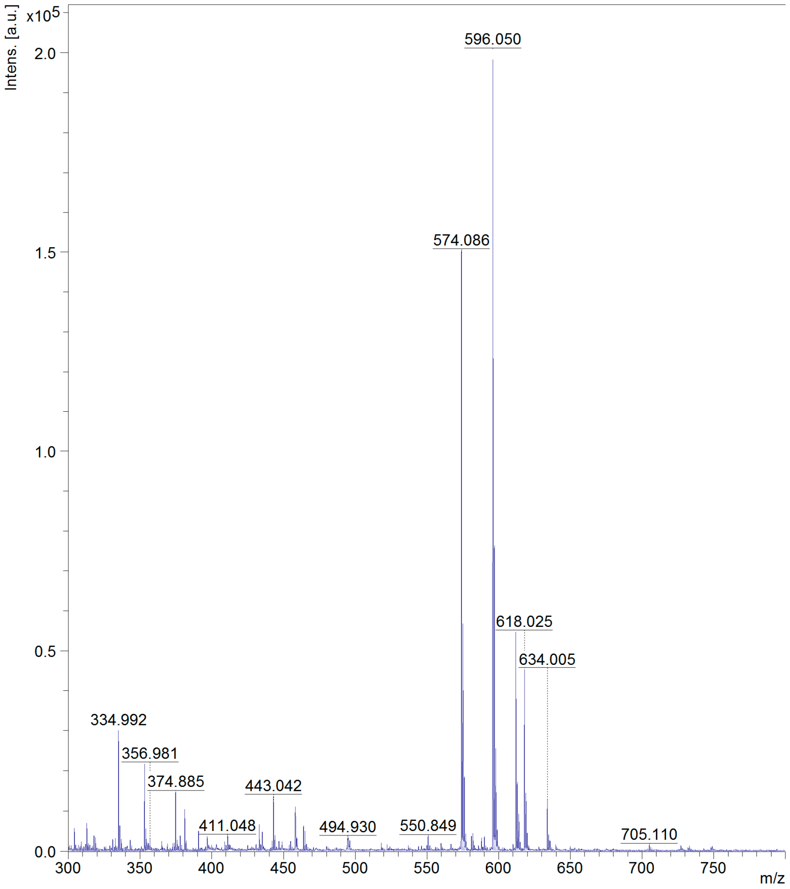

OGF calculated signals 574.23 [M+H]$^+$; 596.21 [M+Na]$^+$; 612.19 [M+K]$^+$
OGF found signals 574.08 (intensity 149518, area 54534); 596.05 (intensity 200743, area 71782); 612.05 (intensity 55496, area 19375);

OGF with Met(O) calculated signals 590.22 [M+H]$^+$; 612.20 [M+Na]$^+$; 628.18 [M+K]$^+$
OGF with Met(O) found signals 590.08 (intensity 2981, area 833); 612.05 (intensity 55496, area 19375)

(F)

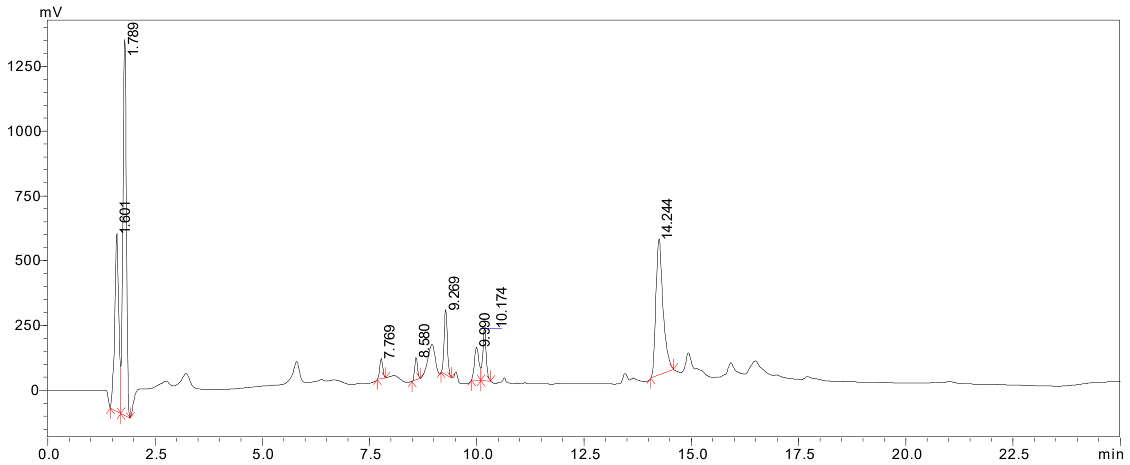

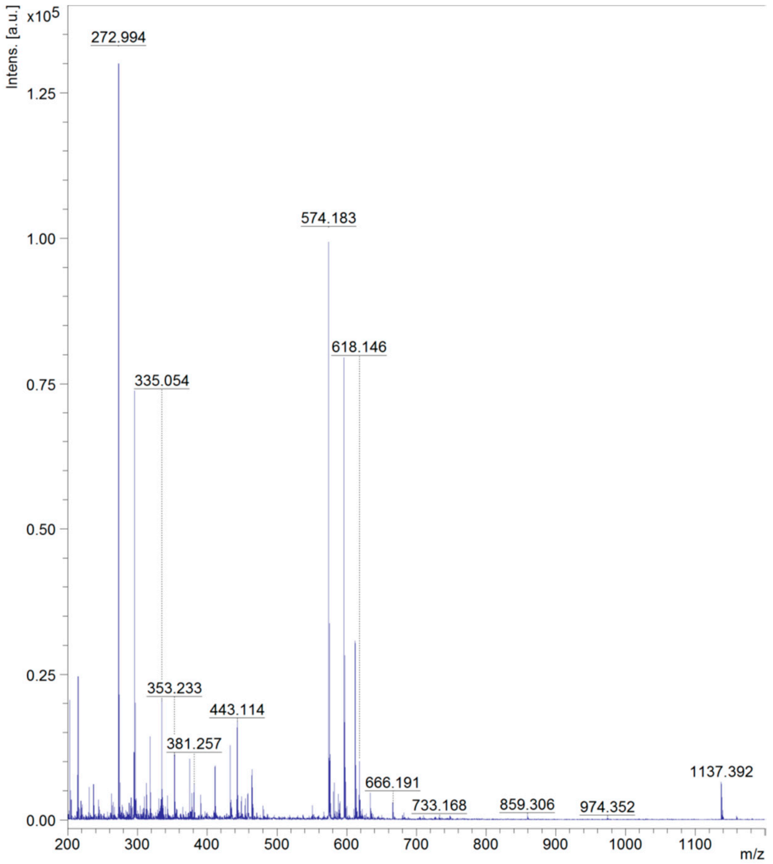

OGF calculated signals 574.23 $[M+H]^+$; 596.21 $[M+Na]^+$; 612.19 $[M+K]^+$
OGF found signals 574.18 (intensity 101730, area 25177); 596.16 (intensity 82039, area 19146); 612.14 (intensity 30802, area 6961);

OGF with Met(O) calculated signals 590.22 $[M+H]^+$; 612.20 $[M+Na]^+$; 628.18 $[M+K]^+$
OGF with Met(O) found signals 590.17 (intensity 2832, area 542); 612.14 (intensity 30802, area 6961); 628.13 (intensity 879, area 180)

OGF without *N*-terminal Tyr calculated signals 411.16 $[M+H]^+$; 433.14 $[M+Na]^+$; 449.12 $[M+K]^+$
OGF without *N*-terminal Tyr found signals 411.13 (intensity 8887, area 1678); 433.10 (intensity 12981, area 2370); 449.08 (intensity 3840, area 800);

Supplementary Figure S4. HPLC chromatograms and MS spectra obtained for OGF before (**A**) and after incubation with medium (DMEM), supplemented with 10% FBS immediately after the start of incubation (**B**), 30 min (**C**), 90 min (**D**), 180 min (**E**), and 23 hours (**F**). Linear gradient 10-90% phase B, 20 min., 1 ml/min, column Kinetex 5 µm XB-C18 100Å 150 x 4.6 mm, 214 nm.

(A)

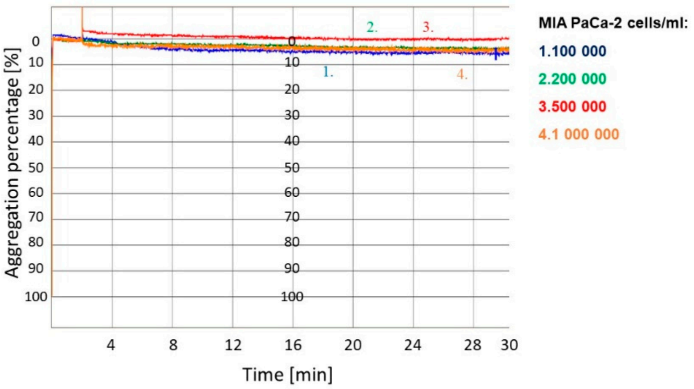

(B)

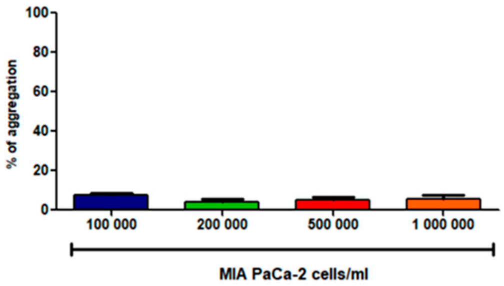

Supplementary Figure S5. Effect of pancreatic cancer cells MIA PaCa-2 on platelets aggregation (TCIPA). **(A)** Representative LTA graph showing TCIPA induced by MIA PaCa-2 cells depending on the concentration. **(B)** Data are mean ± SD of 5 separate determinations.
